# Targeted delivery of a phosphoinositide 3‐kinase γ inhibitor to restore organ function in sepsis

**DOI:** 10.15252/emmm.202114436

**Published:** 2021-09-02

**Authors:** Adrian T Press, Petra Babic, Bianca Hoffmann, Tina Müller, Wanling Foo, Walter Hauswald, Jovana Benecke, Martina Beretta, Zoltán Cseresnyés, Stephanie Hoeppener, Ivo Nischang, Sina M Coldewey, Markus H Gräler, Reinhard Bauer, Falk Gonnert, Nikolaus Gaßler, Reinhard Wetzker, Marc Thilo Figge, Ulrich S Schubert, Michael Bauer

**Affiliations:** ^1^ Department of Anesthesiology and Intensive Care Medicine Jena University Hospital Jena Germany; ^2^ Medical Faculty Friedrich Schiller University Jena Jena Germany; ^3^ Center for Sepsis Control and Care Jena University Hospital Jena Germany; ^4^ Research Group Applied Systems Biology Leibniz Institute for Natural Product Research and Infection Biology ‐ Hans Knoell Institute Jena Germany; ^5^ Leibniz Institute of Photonic Technology Jena Germany; ^6^ Jena Center for Soft Matter (JCSM) Friedrich Schiller University Jena Jena Germany; ^7^ Laboratory of Organic and Macromolecular Chemistry (IOMC) Friedrich Schiller University Jena Jena Germany; ^8^ Septomics Research Centre Jena University Hospital Jena Germany; ^9^ Institute of Molecular Cell Biology Jena University Hospital Jena Germany; ^10^ Section of Pathology Institute of Forensic Medicine Jena University Hospital Jena Germany; ^11^ Institute of Microbiology Faculty of Biological Sciences Friedrich Schiller University Jena Germany

**Keywords:** cholestasis, drug delivery, liver failure, PI3K, sepsis, Microbiology, Virology & Host Pathogen Interaction

## Abstract

Jaundice, the clinical hallmark of infection‐associated liver dysfunction, reflects altered membrane organization of the canalicular pole of hepatocytes and portends poor outcomes. Mice lacking phosphoinositide 3‐kinase‐γ (PI3Kγ) are protected against membrane disintegration and hepatic excretory dysfunction. However, they exhibit a severe immune defect that hinders neutrophil recruitment to sites of infection. To exploit the therapeutic potential of PI3Kγ inhibition in sepsis, a targeted approach to deliver drugs to hepatic parenchymal cells without compromising other cells, in particular immune cells, seems warranted. Here, we demonstrate that nanocarriers functionalized through DY‐635, a fluorescent polymethine dye, and a ligand of organic anion transporters can selectively deliver therapeutics to hepatic parenchymal cells. Applying this strategy to a murine model of sepsis, we observed the PI3Kγ‐dependent restoration of biliary canalicular architecture, maintained excretory liver function, and improved survival without impairing host defense mechanisms. This strategy carries the potential to expand targeted nanomedicines to disease entities with systemic inflammation and concomitantly impaired barrier functionality.

## Graphical abstract

Dye‐functionalized liposomes allow delivery of a PI3K‐γ inhibitor to hepatocytes to resolve sepsis‐related liver failure without ‘off‐target’ effects on immunity.

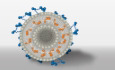

**The paper explained**

**Problem**

Excretory liver failure is associated with poor outcomes in life‐threatening infections. Phosphoinositide 3 kinase (PI3K) γ, a kinase with a critical role in neutrophil chemotaxis and bacterial killing, plays a pivotal role in developing liver failure. However, cell‐type‐specific expression and function of PI3Kγ in the liver is unknown.

**Results**

Here, we demonstrate the expression of PI3Kγ in hepatocytes, bile ducts, and larger vessels in human and mouse liver in addition to the expected high expression in infiltrating neutrophils. Furthermore, dye‐functionalization of liposomes loaded with the PI3Kγ inhibitor AS605240 allows hepatocellular enrichment of the inhibitor while avoiding its uptake in immune cells. This strategy strongly diminished off‐target effects on immunity while preserving the protective effects of PI3Kγ inhibition on canalicular structure and function in hepatocytes.

**Impact**

Nanoformulated therapy carries the potential to restore key cellular functions, such as PI3Kγ signaling with the required cell‐, tissue‐, or organ‐specificity. These novel strategies might allow stopping the domino‐like damage to remote organs in sepsis and offer alternatives beyond the currently available strictly supportive therapeutic options.

## Introduction

Sepsis afflicts approximately 1.7 million adults each year in the United States alone and contributes up to 270,000 in‐hospital deaths (Prescott & Angus, 2018). The current “Sepsis‐3” definition shifted the conceptual focus away from an exaggerated systemic inflammatory response toward abreaction by the host's defense mechanisms affecting both directly involved and remote organs (Fernando *et al*, 2018). In this regard, metabolic (mal)adaptation appears to be of crucial significance (Weber *et al*, 2012; Weis *et al*, 2017).

An improved understanding of clinical and biological interactions that can delineate disease phenotypes is key to identifying successful therapeutic approaches. Among the four clinical phenotypes described by Seymour *et al* (2019) the phenotype characterized by impaired hepatic excretory function was associated with the worst outcomes.

We previously demonstrated that mice lacking PI3Kγ were protected against hepatic excretory dysfunction (i.e., disturbed phase I and II metabolism and transcellular transport) during sepsis (Recknagel *et al*, 2012), yet a concurrent and severe immune defect rendered them vulnerable toward peritonitis. The immune deficiency presumably reflects the critical role of PI3Kγ in neutrophil migration (Hirsch *et al*, 2000). Thus, conflicting functions of PI3Kγ in liver parenchyma and immune cells regarding the net outcome of inhibition might be hypothesized. PI3Kγ expression by cells other than neutrophils has received attention (Patrucco *et al*, 2004) but has not been explored in hepatocytes. Unequivocally, the ability to successfully target PI3Kγ to specific (parenchymal) cells while avoiding potentially deleterious effects on immune function would offer a fundamental improvement in therapeutic interventions for sepsis, a condition where multiple adjunct therapies have failed over decades (Marshall, 2014; Cavaillon *et al*, 2020).

Nanomedicine holds great promise but requires the design of therapeutics with a distinct combination of features such as low toxicity, simplicity, and efficacy (Guidolin & Zheng, 2019). The pharmacology and toxicology of nanocarriers depend on size, composition, surface charge, and shape; these all determine uptake, immune cell recognition, and circulation time. Conversely, an enhanced permeability and retention (EPR) effect leads to passive enrichment of the cargo‐carrier systems in tissues where the barrier function is locally altered (e.g., by tumor or local inflammation), reflecting a significant hurdle for conditions such as sepsis, severe tissue trauma or hemorrhagic shock where barriers are ubiquitously disturbed (Huber‐Lang *et al*, 2018; Liu *et al*, 2020). Modifying nanocarriers with dyes known to act as ligands of organic anion transporters may enable substantial enrichment of the drug cargo into hepatocytes while, at the same time, reducing the uptake by circulating neutrophils or tissue macrophages (e.g., liver Kupffer cells) lacking these transporters (Press *et al*, 2014).

In the current investigation, we aimed to restore liver excretory function by local targeting of PI3Kγ. We hypothesized that selective hepatocellular targeting of PI3Kγ attenuates liver failure in peritonitis‐induced sepsis to minimize side effects on immune function enabling an unimpaired peritoneal host response. To test this postulate, we used dye‐functionalized liposomes to deliver AS605240, a PI3Kγ inhibitor, to hepatic parenchymal cells in a model of severe polymicrobial peritonitis where side effects compromising immunity would be unwelcome.

## Results

### Expression of PI3Kγ in human liver and effect of PI3Kγ null mice on sepsis survival

To scrutinize the cell‐type‐specific involvement of PI3Kγ in sepsis‐induced hepatic excretory dysfunction and characterize the protein as a therapeutic target, expression of the signaling protein in murine and human liver tissue was first investigated. We used a validated polyclonal antibody raised from an orthologue peptide sequence of PI3Kγ in mice and humans (Inprasit *et al*, 2020). Liver tissue slices were prepared from nine patients with very mild to severe liver pathologies, including non‐alcoholic fatty liver disease, liver cirrhosis, and acute liver failure (Appendix Table S1).

**Figure 1:**
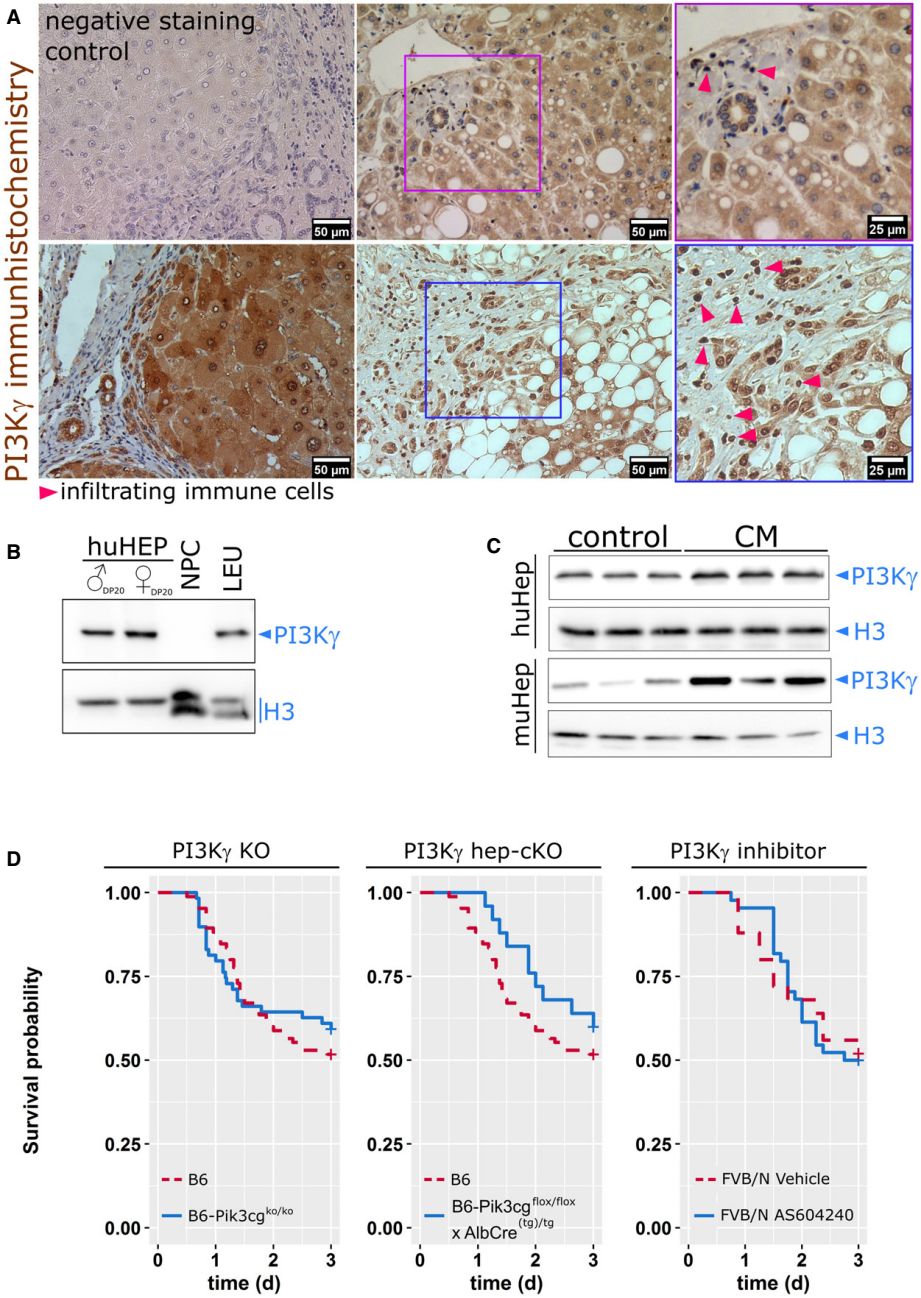
Cell‐type‐specific expression pattern of PI3Kγ in the liver, induction, and function under inflammatory conditions PI3Kγ is expressed by human hepatocytes and infiltrating immune cells in biopsies from patients with minimal to mild inflammatory activity. Triangles point to immune cells (clusters), including some neutrophils known to express PI3Kγ highly. In the negative control, the primary antibody was replaced by an equal volume buffer. The number of included patients, gender, and diagnosis are summarized in Appendix Table S1.PI3Kγ expression in human primary hepatocytes from 20 male (♂) or female (♀) donor pools (HEP, DP20), but not non‐parenchymal cells (NPC). Isolated human leukocytes from healthy volunteers (LEU) served as positive controls. The characterization of the donor pools is summarized in Appendix Table S2.Primary murine hepatocytes and HepG2 cells expressed PI3Kγ under basal conditions; expression was increased at 24 h after stimulation with LPS, IFN‐γ, IL‐1β, and TNF‐α (CM).Survival of WT, PI3Kγ null (left) and liver‐specific PI3Kγ knockout mice (PI3Kγ flox^tg/tg^ × AlbCre^(tg)/tg^, middle) or systemic application of the PI3Kγ inhibitor, AS605240 (right) in a model of polymicrobial sepsis induced by peritoneal contamination with a human stool suspension. Animal numbers: B6: 86 (%male: 52, %female: 48), B6‐Pi3kcg^ko/ko^: 59 (%male: 51, %female 49), B6‐Pi3kcg^flox/flox^ × AlbCre^(tg)/tg^: 25 (%male: 68, %female 32), FVB/N Vehicle: 25 (%male: 44, %female 56), FVB/N AS: 43 (%male: 44, %female 56).

We found consistent staining of PI3Kγ in hepatocytes, bile ducts, and larger vessels in all tissue sections. The intensity of PI3Kγ expression was expectedly high in infiltrating immune cells, primarily neutrophils found within the sections (Fig 1A). Other non‐parenchymal liver cells, in particular Kupffer cells and liver sinusoidal endothelial cells, were negative for PI3Kγ. To validate these results, we performed Western blots with the same antibody against PI3Kγ in a sample of human non‐parenchymal cells (NPCs) and primary human hepatocytes obtained from a pool of 20 male or 20 female donors. In line with the immunohistochemistry staining of patient biopsies, Western blots revealed expression of PI3Kγ in primary human hepatocytes and leukocytes obtained from healthy volunteers. At the same time, liver non‐parenchymal cells, i.e., sinusoidal endothelial cells, Kupffer cells, and cholangiocytes were negative for the signaling protein (Fig 1B). Expression of PI3Kγ was similarly observed in murine hepatocytes and further enhanced in primary human hepatocytes and murine hepatocytes after stimulation with a mix of pro‐inflammatory cytokines (LPS, IFN‐γ, IL‐1β, and TNF‐α) for 24 h indicating involvement within the inflammatory response of these cells (Fig 1C).

We next investigated the effect of systemic and tissue‐specific knockout of PI3Kγ on survival in our murine sepsis model. As shown in Fig 1D, no significant difference in wild‐type and PI3Kγ knockout mice survival rates were observed. Septic PI3Kγ knockout mice did not develop excretory liver failure (Recknagel *et al*, 2012), whereas the liver‐specific, but not the systemic PI3Kγ knockout treatment, resulted in a trend toward increased survival in the initial phase of sepsis (Fig 1D). Knockout of PI3Kγ deleted kinase activity and kinase‐independent functions that, for example, contribute to the integrity of the hepatocyte canalicular membrane (Zucchetti *et al*, 2011). Together, these findings motivated us to attempt hepatocyte‐specific pharmacological selective targeting of the kinase function of PI3Kγ using a small molecular inhibitor of kinase activity as a therapeutic concept.

### Characterization and pharmacological properties of dye‐functionalized lipid nanoparticles for hepatocellular drug delivery

**Figure 2:**
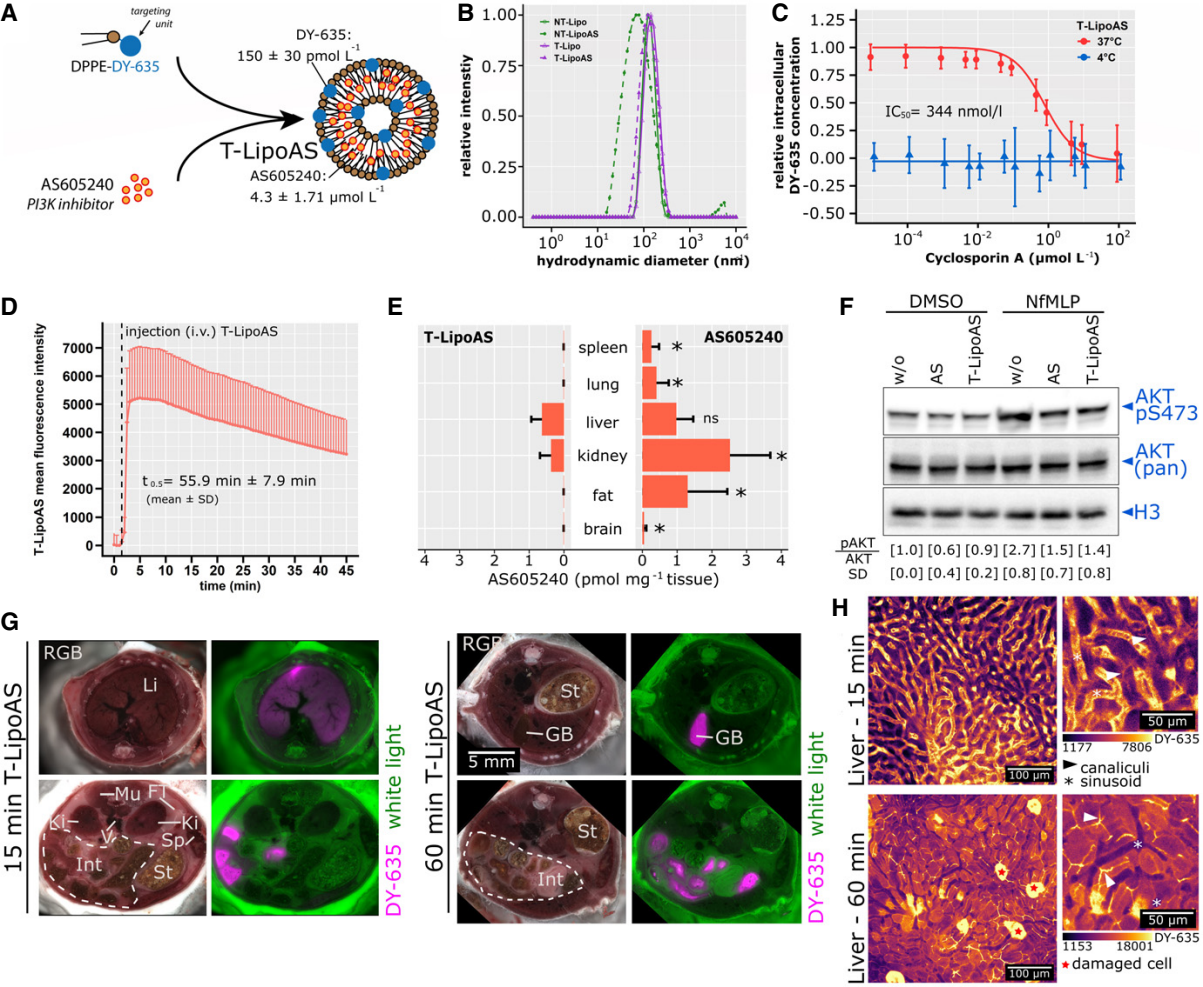
Formulation of targeted liposomes, *in vitro* uptake characteristics, and *in vivo* pharmacokinetic and pharmacodynamic properties Scheme for the preparation of DY‐635 targeted liposomes containing the PI3Kγ inhibitor AS605240 (T‐LipoAS). LC‐MS determined concentrations of AS605240 and DY‐635 individually prepared batches used for all subsequent experiments. The graph depicts the mean ± SD from five different batches.Hydrodynamic diameters of different liposomes in histidine buffer were analyzed by dynamic light scattering. The graph depicts representative plots obtained from dynamic light scattering in Histidine buffer. Further, all batches in this study have been analyzed for size by the manufacturer using DLS and were confirmed to be stable in solution for at least one year.Uptake characteristics of T‐LipoAS by primary human hepatocytes demonstrate competitive inhibition by cyclosporin A‐ and energy dependence, confirming active uptake by organic anion transporters (OATPs). The data points are depicted mean ± SD from two batches of primary human hepatocytes in quadruples.T‐LipoAS plasma disappearance rate in murine ear veins and estimated half‐life obtained by intravital microscopy. The mean fluorescence intensity averaged between the remaining 24 regions of interest (from *n* = 4 FVB/N mice) and the positive standard error depicted in the figure.Quantification of AS60524060 min after injection of 1 mg/kg AS605240 (i.p. in DMSO, *n* = 4 animals) or 2.5 mg/kg T‐LipoAS (i.v. *n* = 5 animals) in tissue lysates quantified by LC‐MS. Bars depict the mean + SD for each group. *P < 0.05 for Wilcoxon test between T‐LipoAS and AS605240 in each tissue.T‐LipoAS effectively inhibits fMLP‐stimulated PI3Kγ, reflected by inhibiting AKT S473 phosphorylation activity in hepatocytes from three different batches. The mean pAKT/AKT ratio and standard deviation (SD) were calculated for all experiments separately.Episcopic imaging of mice treated with T‐LipoAS for 15 or 60 min and relative concentration of the targeting moiety DY‐635 in the different compartments.Intravital microscopy to monitor T‐LipoAS kinetics in the liver. Liposomes appear in the sinusoidal vasculature after 5–15 min and accumulate within hepatocytes. After 35–60 min, the bile canalicular system appears stained, indicating the processing and elimination of the targeting moiety (DY‐635) from T‐LipoAS. Similar distribution patterns had been observed in all repetitions (*n* = 7).

The thiazolidine compound AS605240 was selected as a specific inhibitor of PI3Kγ enzymatic activity known to inhibit neutrophil migration and function in a dose‐dependent manner (Camps *et al*, 2005; Schreiber *et al*, 2010). To specifically direct the drug to liver parenchyma, we used liposomes containing 1,2‐dipalmitoyl‐sn‐glycerol‐3‐phosphoethanolamine (DPPE) azide modified with the fluorescent dye DY‐635. DY‐635 was recently characterized as a molecule specifically recognized and eliminated by hepatocytes (Press *et al*, 2014). DY‐635 conjugated with DPPE was then used to prepare targeted liposomes that deliver the PI3Kγ inhibitor AS605240 into hepatocytes. The liposomes used in this study (five batches) contained 4.3 ± 1.71 µmol/l AS605240 and 130 ± 30 pmol/l DY‐635 (mean ± SD; Fig 2A). All liposomes revealed long‐term stability with mean hydrodynamic diameters (dynamic light scattering (DLS), intensity mode) of 150–160 nm and polydispersity index (PDI) of 0.2. Only non‐targeted, AS605240‐loaded liposomes (NT‐LipoAS) were smaller (average ˜ 90 nm) and had a lower PDI of 0.1 (Fig 2B). The determination of the diameter from cryo‐transmission electron microscopy (cryo‐TEM) images from different liposomal preparations confirmed the hydrodynamic diameters from the DLS analysis. For the AS605240‐loaded liposomes (T‐LipoAS and NT‐LipoAS) and the non‐targeted AS605240‐free liposomes, the diameter was smaller than the hydrodynamic diameter obtained from DLS. This difference could have resulted from variations in the hydration of the different carriers. The DLS of the drug‐free carrier, T‐Lipo, revealed a slightly higher diameter (190 nm). Quantification of the diameters by transmission electron microscopy confirmed the measurements obtained by DLS (Appendix Fig S1).

Liposomes were further characterized by analytical ultracentrifugation (Cinar *et al*, 2020). Liposomes did not sediment at a measured fluid density (ϱ_0_) of 1.0822 g/cm^3^ and dynamic viscosity (η_0_) of 1.1996 mPas of a heavy water/buffer mixture, suggesting a relatively high partial specific volume (ν) of ˜ 0.924 cm^3^/g. The sedimentation velocity experiments suggest a hydrodynamic diameter of ˜ 150 nm (for T‐Lipo) and 141 nm (for T‐LipoAS) when extrapolating the sedimentation coefficient to infinite dilution. These values are slightly smaller than the hydrodynamic diameters obtained by DLS. Liposomes diluted in the buffer showed stability during sedimentation at 10,000 rpm and redispersibility by simple shaking of the sedimented material (Appendix Fig S2).

DY‐635 facilitates targeted delivery of nanocarriers to hepatocytes and specific tumor cells expressing organic anion transporters (OATs) and OAT pumps (OATPs) (Press *et al*, 2014; Ernst *et al*, 2020; Shkodra *et al*, 2020). To determine whether the targeting unit on liposomes improves hepatocyte uptake, we used transporter‐qualified primary human hepatocytes expressing various OATs and OATPs, among other basolateral hepatocellular uptake transporters. Incubation with T‐LipoAS at 37°C led to a substantial uptake diminished by ciclosporin A, which acts as a competitive inhibitor of OAT(P)s. The calculated inhibitory concentration (IC)_50_ of 344 nmol/l was similar to a previously reported value for DY‐635‐conjugated polymer nanoparticles (Press *et al*, 2014). The ciclosporin A‐sensitive uptake was quenched by lowering the temperature to 4°C, indicating the active and OAT(P)‐mediated uptake process (Fig 2C).

The intravenous injection of T‐LipoAS and following plasma decay was analyzed by intravital microscopy of the DY‐635 targeting unit in murine ear veins. The linear regression in the decay phase resulted in an estimated half‐life of 55.9 min ± 7.9 min (Fig 2D). Next, we analyzed tissue distribution of AS605240 in mice at 60 min post‐injection of free AS605240 (4 mg/kg, intraperitoneal) or T‐LipoAS (2.5 mg/kg, intravenous). The doses were matched based on preliminary experiments to achieve comparable concentrations within the liver. Free AS605240 injected intraperitoneally accumulated in all analyzed tissues (spleen, lung, liver, kidney, fat, and brain), while AS605240 derived from T‐LipoAS was only found in the liver and kidney. The concentrations of AS605240 accumulated in the liver at 60 min after injection did not significantly differ between formulations (Fig 2E), confirming enriched accumulation of the compound in the liver while avoiding other organs except the kidney. AS605240 and its nanoformulation inhibit PI3Kγ‐mediated AKT serine (S)473 phosphorylation induced by *N*‐formylmethionine‐leucyl‐phenylalanine (fMLP) was investigated in primary human hepatocytes. These results confirm the delivery of AS605240 and the inhibition by the liposomal formulation (Fig 2F).

This notion was confirmed by episcopic imaging detecting DY‐635 fluorescence from injected T‐LipoAS to investigate the biodistribution of the carrier. As early as 15 min post‐administration of T‐LipoAS, there was a marked increase in DY‐635 signal intensity in liver tissue and, after hepatobiliary excretion, in proximal parts of the small intestine. By contrast, little to no DY‐635 fluorescence signal was contributed by the spleen or lung. After 1 h, DY‐635 was mainly eliminated through the hepatobiliary system as indicated by the stained gall bladder and gastrointestinal lumen (Fig 2G).

The liver consists of various cell types controlling metabolism and the immune response. Kupffer cells (the liver macrophage population) and liver‐specific endothelial cells (LSECs) have been shown to sequester nanocarriers (Press *et al*, 2014; Tavares *et al*, 2017; Campbell *et al*, 2018). Both cell types would be considered “off‐target” for our delivery strategy since they strongly contribute to the immune response against invading microorganisms. Therefore, cell‐type‐specific targeting of loaded liposomes within the liver was further investigated by time‐lapse intravital microscopy (Fig 2H). This method enables differentiation of parenchymal (hepatocytes) and non‐parenchymal (Kupffer cells, LSECs) within the liver due to their different shape, location, and NAD(P)H fluorescence. After intravenous injection, T‐LipoAS started to appear in the capillary system (sinusoids; Fig 2H). T‐LipoAS then rapidly accumulated within hepatocytes. Clearance of nanocarriers by Kupffer cells or LSECs can be identified with this method via monitoring of line‐shaped patterns or spots at the interphase between NADPH‐positive hepatocytes and the sinusoids (Guidolin & Zheng, 2019; Cinar *et al*, 2020). No signal of T‐LipoAS accumulated at these locations. The appearance of line structures within the hepatocytes indicates the accumulation of DY‐635 in bile canaliculi, the microscopic structure formed by hepatocytes within the bile drainage pathway, further confirming the hepatobiliary elimination of DY‐635. The apparent absence of DY‐635 fluorescence associated with non‐parenchymal cells in the liver in combination with the appearance of DY‐635 derived from T‐LipoAS within hepatocytes and canaliculi indicates a highly selective uptake and processing of targeted nanocarriers through hepatocytes.

**Figure 3:**
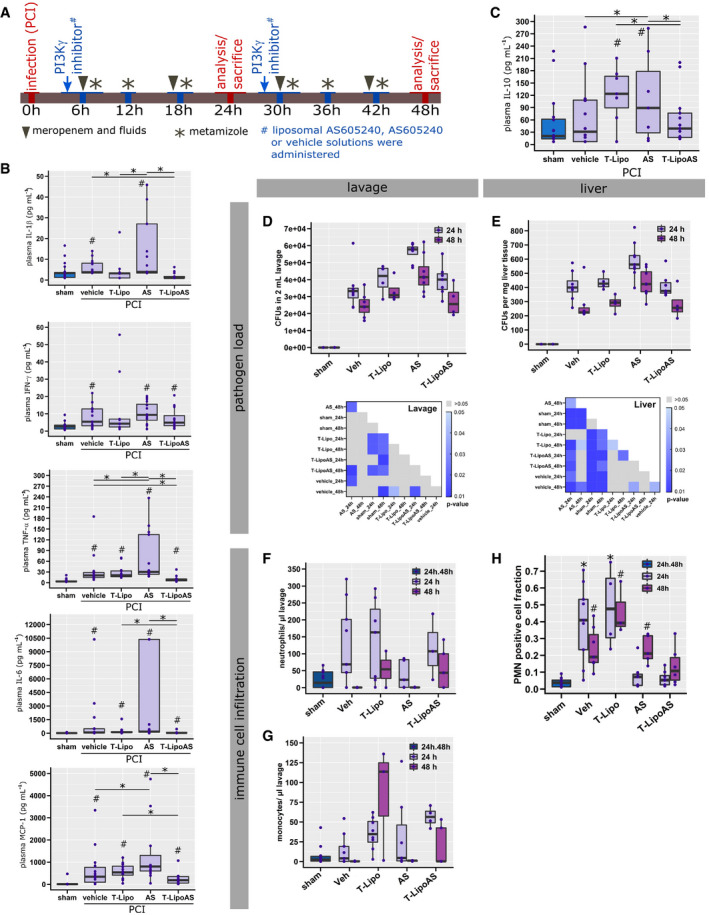
Targeted delivery of AS605240 prevents untoward effects on the innate immune response to peritonitis AGraphical summary of the murine sepsis model and experimental design.B, CPro‐ (B) and anti‐inflammatory (C) cytokines were analyzed in EDTA‐plasma from peritoneal contamination and infection (PCI) and sham animals. ^#^ *P* < 0.05 against sham, *P < 0.05 as indicated; Kruskal–Wallis ANOVA with controlled false‐discovery rates (Benjamini–Hochberg procedure).D, EColony‐forming units (CFUs) were analyzed from (D) abdominal lavage and (E) liver homogenates. Significance maps depict *P*‐values from a Kruskal–Wallis ANOVA with controlled false‐discovery rates applying the Benjamini‐Hochberg procedure.F, GConcentration of (F) neutrophils and (G) monocytes in 2 ml peritoneal lavage.HInfiltrating polymorphonuclear neutrophils (PMN) were assessed from histological liver sections after immune fluorescent staining. *P < 0.05 against sham, ^#^ *P* < 0.05 against 24 h; Kruskal–Wallis ANOVA with controlled false‐discovery rates (Benjamini‐Hochberg procedure). Data information: (B–H) The data are depicted as box plots with median (line). The box describes the [Q1 = 0.25, Q3 = 0.75] interquartile range (IQR), and whiskers the range as following: Q1–1.5*IQR and Q3 + 1.5 IQR. Each data point depicts the result from an individual animal.

Cell culture models cannot resemble the complexity of the pathophysiological mechanisms of sepsis and cannot provide evidence that the suggested targeting properties of T‐LipoAS are maintained under such circumstances. Therefore, the targeting procedure was tested in a complex rodent model of sepsis based on peritoneal contamination and infection (PCI). In this model, human feces are administered intraperitoneally to induce life‐threatening polymicrobial infection. This model is well characterized with signs of excretory liver failure detectable as early as 6 h after initiation of infection (Gonnert *et al*, 2011; Recknagel *et al*, 2012; Schaarschmidt *et al*, 2018; Sommerfeld *et al*, 2020). To explore the targeting approach, animals were treated with free AS605240 or T‐LipoAS at 6 h after infection with functional assessment at 24 and 48 h. All animals received supportive antibiotic (meropenem) and analgesic (metamizole) therapy (Fig 3A).

Our previous report shows PI3Kγ knockout mice display a pronounced “cytokine storm” in response to severe infection that exceeds levels seen in wild‐type animals (Recknagel *et al*, 2012). Similar effects were observed for non‐targeted pharmacologic inhibition of PI3Kγ by free AS605240 in the present study. Several pro‐inflammatory cytokines (IL‐1α, IL‐1β, IL‐6, IL‐27, IFN‐β, IFN‐γ, TNF‐α, GM‐CSF, and MCP‐1) and the anti‐inflammatory cytokine, IL‐10, were analyzed. In septic animals treated with free AS605240 (PCI, AS group), IFN‐β, TNF‐α, GMCSF, MCP‐1, and IL‐10 levels were significantly elevated in comparison with septic animals treated with vehicle (PCI, Vehicle group). IL‐1α, IL‐1β, IFN‐γ, and IL‐27 trended toward an increase (Fig 3B,C, Appendix Fig S3A). An unsupervised clustering of the animals, performed according to similarities in their cytokine profile, also suggested a “cytokine storm” was present, particularly in septic animals treated with free AS605240 (Appendix Fig S3B). This untoward effect on systemic inflammation was not observed when the inhibitor was formulated within liposomes (T‐LipoAS; Fig 3B, Appendix Fig S3A and B). In contrast to the apparent pro‐inflammatory effect of systemic PI3Kγ inhibition by free AS605240, the absence of the cytokine storm in T‐LipoAS‐treated animals is consistent with specific targeting of the PI3Kγ inhibitor in parenchymal liver cells while sparing the immune cell compartment (Fig 2C).

To further investigate this critical aspect of cell‐type specificity of T‐LipoAS, we examined innate immunity (bacterial clearance and neutrophil recruitment) using functional assays. Most importantly, intraperitoneal‐administered free AS605240 in the PCI‐model resulted in an increased pathogen load within the peritoneal cavity (Fig 3D), the primary site of infection, and in liver tissue (Fig 3E). This was absent when hepatocellular inhibition of PI3Kγ was achieved by hepatocyte‐targeted inhibition with T‐LipoAS. Thus, the increased pathogen load may reflect impaired immune cell recruitment and function after intraperitoneal administration of free AS605240. Consequently, lower concentrations of neutrophils (Fig 3F) and monocytes (Fig 3G) were detected within the peritoneal cavity of animals treated with free AS605240. Restricted phagocyte motility is a well‐known response to pharmacological inhibition of PI3Kγ activity (Camps *et al*, 2005). Together, these data reveal that T‐LipoAS, in contrast to free AS605240, does not affect phagocyte recruitment and bacterial clearance in the peritoneal cavity. Systemic effects of the targeted nanocarrier T‐LipoAS on the immune response of septic mice are missing.

**Figure 4:**
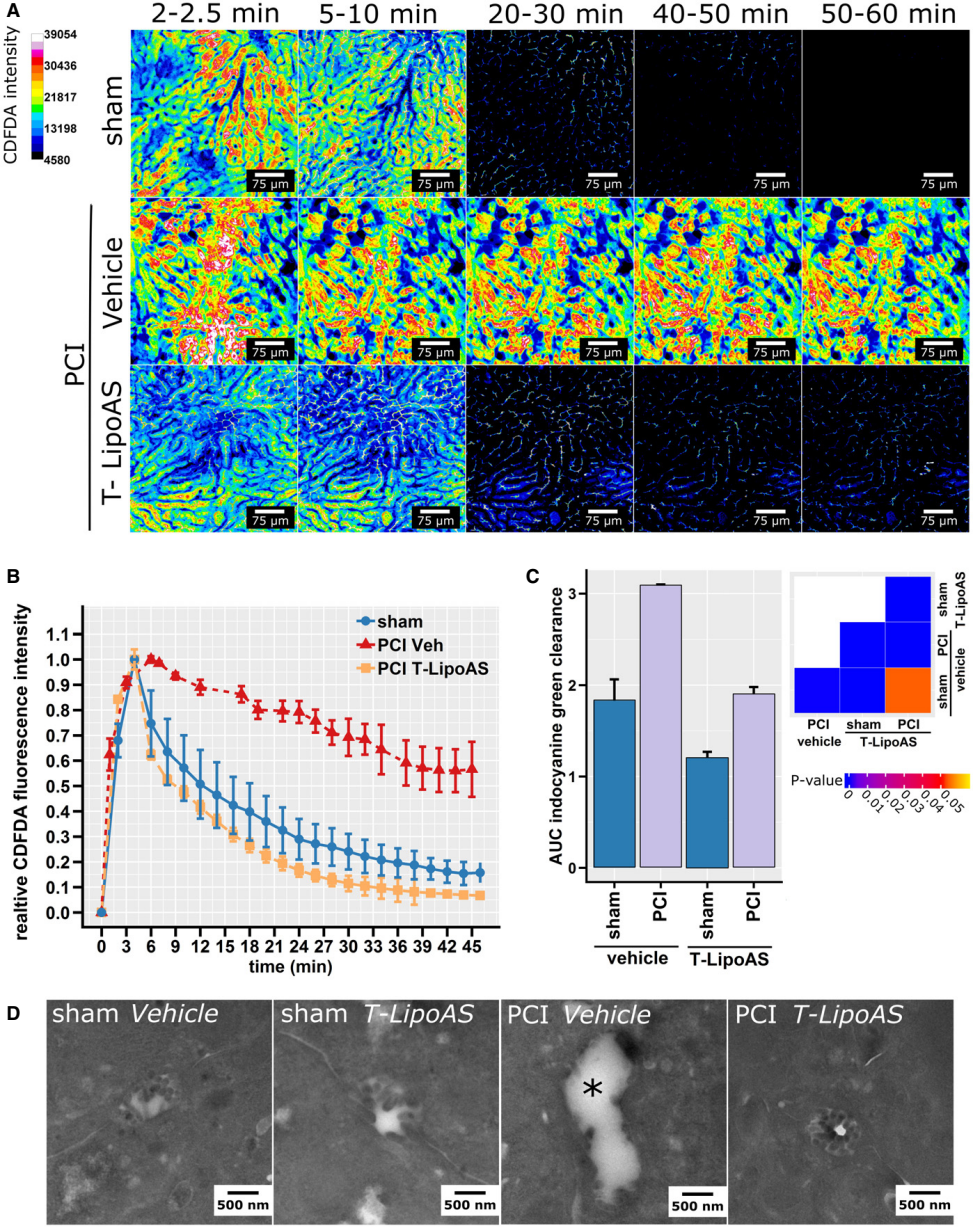
Nanoformulated PI3Kγ inhibition restores excretory liver function *in vivo* Intravital microscopy of elimination of CDFDA, a fluorescent substrate subject to excretory elimination by hepatocytes via Mrp‐2, allows direct visualization of excretory function in sham or septic (PCI) animals treated with vehicle or a hepatocyte‐directed inhibitor of PI3Kγ (T‐LipoAS).Normalized CDFDA intensities quantified from intravital images depict plasma disappearance curves (A, B) Representative intravital images and analysis (mean ± SD) from 5 (sham), 4 (PCI T‐LipoAS), and 3 (PCI Veh) animals. Sham and PCI animals treated with T‐LipoAS showed a classical biphasic decay curve, while PCI vehicles showed a relatively linear decay and a higher area under the curve indicative of excretory liver failure.Similar results were obtained when the liver excretory function was tested by indocyanine green clearance. Indocyanine green was detected non‐invasively by multispectral optoacoustic tomography. The ability to clear ICG was quantitatively analyzed by comparing areas under the curve (AUC). Groups of 9 (sham vehicle), 8 (sham LipoAS), 12 (PCI vehicle). and 15 (PCI LipoAS) animals were analyzed. Bars depict mean + SD.Representative transmission electron micrographs from bile canaliculi of healthy control (sham) and septic animals (PCI) treated with vehicle or the PI3Kγ inhibitors, free AS, or T‐LipoAS. The asterisk marks a bile canaliculus where typical brush borders are lost due to sepsis.

Backing these findings, assays for pathogen load in liver homogenates likewise disclosed differential effects of T‐LipoAs compared with free AS605240. As shown in Fig 3E, the bacterial burden in the liver of animals treated with T‐LipoAS was equal to vehicle control and significantly lower than after treating septic mice with the free PI3Kγ inhibitor. AS605240 formulated into a liposomal carrier system appears to prevent the migration‐inhibiting effect of AS605240 on phagocyte recruitment and the resultant failure to clear bacteria. Notwithstanding, hepatic parenchymal infiltration of neutrophils in both AS and T‐LipoAS treated septic animals was significantly reduced compared to vehicle controls (Fig 3H). Whereas inhibition of neutrophil motility by applying free AS605240 fits the observations in the peritoneal cavity, the reduced neutrophil accumulation in the liver of T‐LipoAS treated mice remains elusive. Diminished neutrophil accumulation in the liver might reflect a protective effect on hepatocyte function T‐LipoAS (Fig 4) since inflamed and damaged liver parenchyma is known to release damage‐associated molecular patterns (DAMPs), which provoke efficient attraction of neutrophils (Marques *et al*, 2012; Pittman & Kubes, 2013). Hence, restoration of septic liver function induced by T‐LipoAS may be accompanied by a diminished release of DAMPs and correspondingly reduced infiltration of neutrophils. Our data collectively corroborate the regenerative effects of T‐LipoAS on liver parenchyma and unveil the lack of suppressive effects of T‐LipoAS on systemic anti‐infective immune responses in septic mice.

As mentioned above, and based on previous observations in PI3Kγ knockout mice (Recknagel *et al*, 2012), we hypothesized that the protective effect of hepatocyte‐targeted PI3Kγ‐inhibition was due to preservation of excretory liver function. Three different approaches were used to investigate the effects of T‐LipoAS on excretory liver failure after 24 h of sepsis (Fig 3A). First, intravital microscopy was applied to assess accumulation and clearance of the Mrp2 substrate, CDFDA, as a surrogate for the excretory function of endogenous substrates of this pathway, e.g., bilirubin (Fig 4A and B). CDFDA in control (sham animals, injected i.p. with sterile saline) accumulated rapidly in the liver, specifically in hepatocytes, but was effectively eliminated within 60 min. However, in sepsis (PCI), CDFDA accumulated as rapidly as in the reference group, but elimination was visibly prolonged, as reflected by a change in elimination kinetics. Intriguingly, treatment with T‐LipoAS fully resolved the effects of sepsis on liver excretory function; the initial accumulation and elimination were similar to that seen in non‐infected sham animals (Fig 4B). To further validate these findings, we investigated the plasma disappearance rate of indocyanine green (ICG), a clinically approved dye used to assess excretory liver function. Plasma disappearance is analyzed in patients by pulse densitometry, but such devices are lacking in small rodents. Due to the (near)infrared (NIR) absorbance of ICG above 750 nm, multispectral optoacoustic tomography can be employed, allowing non‐invasive detection of NIR at up to video frame rate and with good spatial resolution. Applying automated image analysis algorithms, we identified suitable areas for quantification of ICG clearance over time in each animal and investigated elimination through quantification of the area under the curve (AUC; Appendix Fig S4). The increased AUC reflects decreased elimination of ICG from plasma and correlates with increased hepatic ICG retention, i.e., lowered elimination capability. Similar to CDFDA clearance, ICG also accumulated significantly in animals at 24 h post‐induction of sepsis (Fig 3C). This effect was also prevented by a single injection of liposomal AS605240 (T‐LipoAS) for hepatocyte‐targeted PI3Kγ inhibition at 6 h.

The loss of the ability of hepatocytes to eliminate endo‐ and xenobiotics, as probed by the dye‐clearance assays, was reflected by structural changes such as the loss of canalicular brush borders. In line with previous reports, most canalicular brush borders were lost at 24 h, while these structures remained intact in mice treated with the nanoformulated PI3Kγ inhibitor AS605240 (T‐LipoAS), similar to our previous observation in PI3Kγ knockout mice (Fig 4D) (Recknagel *et al*, 2012). Taken together, these findings demonstrate morphological and functional protection of hepatocytes by treatment with the PI3K inhibitor AS605240 in a hepatocyte‐targeted carrier.

**Figure 5:**
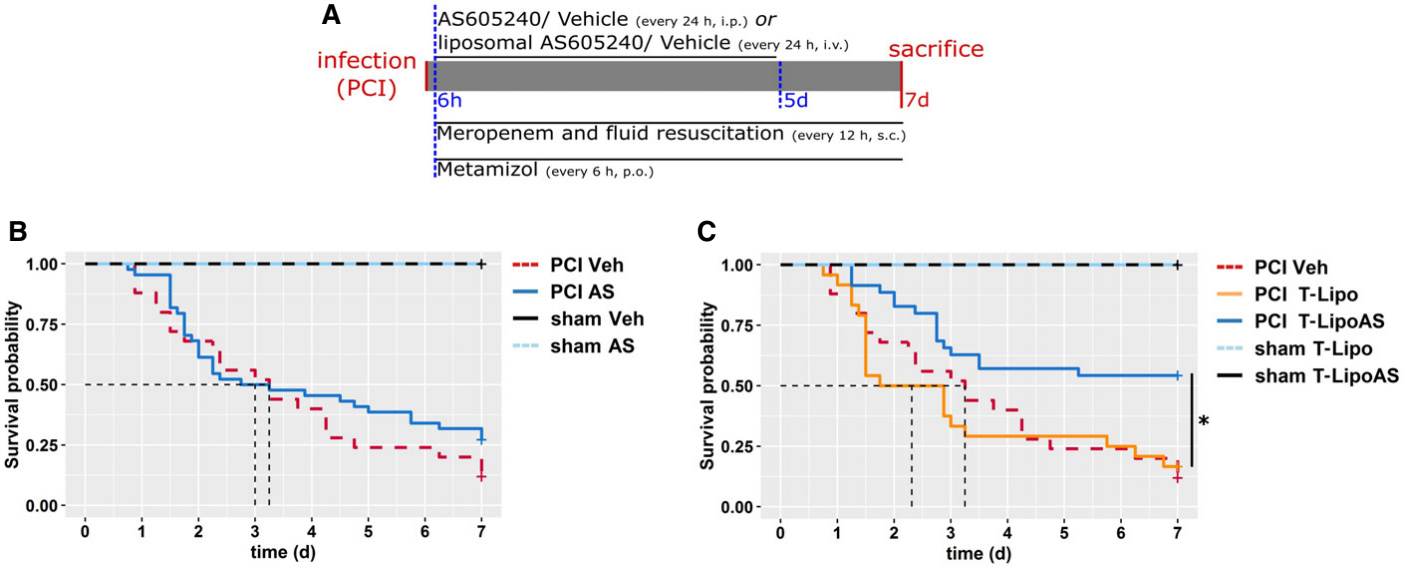
Restoration of excretory liver function in sepsis is associated with improved survival ATreatment scheme and adjunctive therapy in the survival experiment. All animals received meropenem (25 mg/kg body weight, s.c.) twice daily and metamizole (2.5 mg per animal, p.o, every 6 h).B, CSurvival analysis of PCI and sham animals. Subgroups were treated with (B) free AS605240 or vehicle (Veh; i.p.) or (C) with liposomal formulated AS605240 (T‐LipoAS) or T‐Lipo (i.v.) as the specific vehicle control once per day. *P < 0.05, log‐rank test. Animal numbers: PCI Veh: 25 (%male: 44, %female: 56), PCI AS: 43 (%male: 44, %female: 56), PCI: T‐Lipo 24 (%male: 50, %female: 50), PCI‐T‐LipoAS: 35 (%male: 49, %female: 51), sham Veh: 7 (%male: 100), sham AS: 7 (%male 57, %female: 43), sham T‐Lipo (%male 25, %female: 75): 4, and sham T‐LipoAS 7 animals (%male 29, %female: 71).

Since sepsis‐associated liver failure is linked with poor survival (Yan *et al*, 2014; Ye *et al*, 2019), targeted delivery of AS605240 through T‐LipoAS improved liver function and overall outcome (Fig 5). The chosen sepsis model aimed to mimic a clinical situation. Animals were infected with a hefty dose of fecal slurry followed by administration of meropenem, a broad‐spectrum antibiotic, to achieve an approximate 50% lethality. In the absence of any surgical procedure to clear bacteria from the abdominal cavity (the primary site of infection), chronic abscesses are formed, making survival analysis beyond 7 days challenging to interpret. Different AS605240 and vehicle formulations were administered daily over the first 5 days, commencing at 6 h post‐infection (Fig 5A). No survival difference was observed between animals treated systemically with AS605240 and septic controls (Fig 5B). AS605240 formulated in non‐targeted liposomes (NT‐LipoAS) even worsened mortality, perhaps due to improved bioavailability and immune‐cell clearing of non‐targeted liposomes compared to the targeted formulations (Appendix Fig S5). By contrast, the DY‐635 formulation, T‐LipoAS, prolonged and significantly increased survival (Fig 5C, Appendix Fig S5), supporting findings that the hepatocyte‐specific delivery of AS605240 was achieved and off‐target effects on the immune system significantly reduced.

## Discussion

Infection‐driven organ dysfunction conveys a significant burden of disease on contemporary intensive care units. While overt liver failure is considered rare and late, moderate metabolic and excretory malfunction is a commonplace event in infectious disease that critically depends on PI3Kγ signaling (Recknagel *et al*, 2012). Typically, PI3Kγ is considered a key “switch” for membrane organization within the immune system, although additional functions in other cells and tissues have been recognized, e.g., in the central nervous or cardiovascular systems (Hirsch *et al*, 2000). To explore the potential separation of the cholestasis‐preventing effect of PI3Kγ inhibition from untoward effects on immunity, we explored the cell‐type‐specific expression pattern of the gene in murine and human liver cells. We found that PI3Kγ is expressed across species in liver parenchymal cells, offering the possibility that selective inhibition of hepatocytes, if achieved, could improve liver function without impairing immune cell function. Experimental data identified downregulation of canalicular transporters in hepatocytes, especially Mrp2 as critical elements of impaired excretory function. Mechanistically, insertion of Mrp2 into “brush borders” of the canalicular membrane is (i) a hallmark of cholestasis, (ii) has been found diminished in sepsis, and (iii) this phenotype depends critically upon PI3Kγ under these conditions (Recknagel *et al*, 2012). However, PI3Kγ is also recognized for its central role in leukocyte recruitment to sites of infection (Hirsch *et al*, 2000; Halbgebauer *et al*, 2018), a prerequisite for pathogen clearance, and thus a successful host response. The systemic inhibition of PI3Kγ and the associated elevation of plasma‐cytokines compared to mice with sepsis may further reduce cytokine gradients between plasma and local tissue concentrations. The reduced cytokine gradient may lower the chemotaxis of immune cells along these cytokine gradients into the abdominal cavity to fight infection, contributing to additional detrimental side effects independent of the PI3Kγ inhibition in those cells (Namas *et al*, 2012; Peng *et al*, 2014).

Consistent with this notion, neither the ubiquitous knockdown of the PI3Kγ gene nor its systemic pharmacological inhibition improved survival even though the knockout prevents hyperbilirubinemia as a reflection of cholestasis (Recknagel *et al*, 2012).

Intriguingly, nanoformulation and targeting of the PI3Kγ inhibitor AS605240 allowed avoidance of untoward extrahepatic effects of PI3Kγ inhibition, for example neutrophils, and improved outcomes in a murine model of sepsis. One can speculate why the effect of the kinase‐inhibitor AS605240 on survival was more pronounced than the hepatocyte‐specific PI3Kγ knockout; a maintained chaperoning function of the protein (Perino *et al*, 2011) may be involved and would argue for a kinase‐inhibitor rather than a gene silencing (siRNA) strategy.

Attempts to deliver small molecular drugs selectively to organs, e.g., hepatic parenchyma, hold considerable promise (Shkodra *et al*, 2020). This is in addition to well‐developed and clinically available strategies for siRNA delivery (Ray *et al*, 2017). Consistent with our previous results in PI3Kγ knockout mice (Recknagel *et al*, 2012), systemic inhibition of PI3Kγ by intraperitoneally administered AS605240 led to an increased and prolonged cytokine storm compared to septic animals treated with the corresponding vehicle, indicative of impaired neutrophil migration and delayed resolution of infection. This cytokine storm was accompanied by a prolonged elevation of tissue damage markers, a significant fall in body weight, and impaired clinical status reflecting significant morbidity. Thus, a protective effect of systemic PI3Kγ inhibition on hepatocellular excretory function appears to be antagonized by untoward side effects, including those acting on the immuno‐inflammatory host response.

By contrast, septic animals treated with T‐LipoAS showed no signs of an impaired immune response at the peritoneal site of infection. Thus, direct targeting of hepatocytes to prevent liver failure appears to preserve the host's ability to fight infection. Neither PI3Kγ knockout nor systemic, non‐targeted application of AS605240 could improve survival. Instead, non‐targeted nanoformulations of AS605240 even led to an increase in mortality, possibly consistent with clearance of non‐functionalized nanoparticles by innate immune cells, thereby potentiating immunosuppressive off‐target effects.

Local, inflammation‐triggered alterations of the endothelial barrier, e.g., in a tumor micro milieu, likely contribute to the EPR effect used for targeted delivery of nanomedicines (Liu *et al*, 2020). It is thus noteworthy that DY‐635 targeting could achieve preferential accumulation of AS605240 within the liver while avoiding off‐target effects on immunity, and this occurred even under conditions of systemic inflammation with presumably widespread altered barrier integrity. The pharmacological effects of T‐LipoAs accumulation in the kidney (Fig 2D) have not been investigated in detail; nevertheless, the molar amounts are substantially lower compared to administration of the “free” drug that is accompanied by approximately 5‐ to 10‐fold higher tissue accumulation in renal tissue (Fig 2D). In light of the significantly improved survival of T‐LipoAs treated septic mice, we have no evidence of impaired kidney function by the drug‐loaded nanocarriers, although the mechanism and significance of accumulation in the kidney warrant further investigation.

As an obvious limitation, our study barely provides a proof of concept for a therapeutic intervention targeting parenchymal cells selectively. The feasible application of DY‐635 mediated targeting of nanoformulated drugs in a disease model beyond our previous report in healthy mice (Press *et al*, 2014), and the here‐documented expression of PI3Kγ in human hepatocytes should prompt, however, further translational studies.

### Conclusion

The present study demonstrates a druggable link between PI3Kγ activity and excretory liver failure in sepsis, a syndrome associated with poor outcomes. To overcome the off‐target effects on immunity associated with systemic PI3Kγ inhibition, we successfully characterized a DY‐635 conjugated liposomal carrier, increasing the bioavailability of the PI3Kγ inhibitor AS605240 compartmentalized to hepatocytes, significantly improving liver function and survival. The simple and straightforward formulation and the described expression of PI3Kγ in human hepatocytes represent a significant opportunity for clinical translation of these carriers to target PI3Kγ‐mediated liver failure with a simultaneous reduction in side effects on the immune system.

## Materials and Methods

### Liposomes

Liposomes were provided by BioLiTec Research GmbH, Jena, Germany. 1,2‐Dipalmitoyl‐sn‐glycerol‐3‐phosphoethanolamine (DPPE) azide was conjugated to DY‐635‐alkin (Dyomics GmbH, Jena, Germany) and used to prepare liposomes using a 100 nm extruder. AS6052040‐loaded liposomes were loaded with 0.2 mg AS605240 per 0.2 mg lipid mixture per ml in histidine buffer.

### AS605240 quantification

AS605240 concentration in liposomes was quantified by high‐performance liquid chromatography coupled to tandem mass spectrometry (LC‐MS/MS). The LC system (Shimadzu) was equipped with a CBM‐20A Communication Bus Module, DGU‐20A5R Degassing Unit, LC‐20AD Prominence Liquid Chromatograph, SIL‐20AC Prominence autosampler, and a CTO‐20AC Prominence Column oven. A triple quadrupole mass spectrometer LCMS‐8050 (Shimadzu) equipped with an APCI source operating in positive mode was used for detection of AS605240 under the following parameters: nebulizing gas flow: 3 l/min, heating gas flow: 10 l/min, interface temperature: 350°C, desolvation line temperature: 250°C, heat block temperature: 200°C, drying gas flow: 10 l/min. In Q1 and Q3, both quadrupoles were operated at a mass resolution medium with a dwell time of 9.0 ms for ergosterol and 6.0 ms for AS605240 (Abmole Bioscience). AS605240 was identified with an *m*/*z* of 258/187, and ergosterol with *m*/*z* 378/69 in multiple reaction monitoring modes. Gradient chromatographic separation was performed on a 2 × 60 mm Multo‐High100 RP‐18 column (CS‐Chromatographie Service, Langerwehe, Germany) with a particle size of 3 µm. The column was tempered at 50°C. Mobile phase A consisted of 0.1% (v/v) formic acid in deionized water and mobile phase B of 100% methanol. The column was equilibrated in 10% B with a flow rate of 0.4 ml/min. The mobile phase was switched to 100% B after sample injection for elution. The flow rate was increased linearly from 0.4 ml/min at 3 min to 0.8 ml/min at 5 min and remained constant until 7 min. Subsequently, the mobile phase was changed to 10% B in one step, and the flow rate was decreased linearly from 0.8 ml/min at 7.8 min to 0.3 ml/min at 8.5 min and remained constant until the end of the elution program at 9.5 min. Samples were diluted 1:10 in methanol, and 3 µM ergosterol (Sigma Aldrich, Germany) was added as an internal standard prior to detection. The injection volume of the sample was 3 µl. The analytical results were quantified with LabSolutions 5.91 (Shimadzu, Japan) based on an external AS605240 standard curve.

### DY‐635 quantification

Liposomes were prepared as described for AS605240 quantification. DY‐635 was detected using the Merck‐Hitachi Elite LaChrom liquid chromatography (LC) system (VWR, Darmstadt, Germany). The LC system was equipped with the L‐7250 autosampler, Merck Peltier sample cooler for L‐7250, L‐2130 liquid chromatograph, the L‐2300 column oven and the L‐2485 fluorescence detector. Chromatographical separation was performed on a 60x2 mm MultoHigh 100 RP 18 column with 3 μm particle size (CS‐Chromatographie Service, Langerwehe, Germany) at a constant temperature of 50°C using the following gradient: The column was equilibrated in 10% B (methanol) and 90% A (0.1% (v/v) formic acid in ddH2O) with a flow rate of 0.4 ml/min. At 0.1 min, the mobile phase switched to 100% B and 0.5 ml/min. The flow rate increased linearly from 0.5 ml/min at 3.5 min to 0.7 ml/min at 6.0 min and from 0.7 ml/min at 6.0 min to 0.8 ml/min at 10 min and remained constant until 13 min. Subsequently, the mobile phase changed to 10% B at 13.1 min, and the flow rate decreased linearly from 0.8 ml/min at 13.5 min to 0.5 ml/min at 15.0 min. Finally, the flow rate decreased to 0.4 ml/min at 15.5 min and remained constant until the end of the program at 16.0 min. DY‐635 eluted from the column at 3.7 min and was detected using 595 nm excitation wavelengths and 670 nm emission wavelength. The injection volume was 10 µl. DY‐635 concentrations were quantified using the EZChrom Elite 3.1.3 software based on an external calibration curve.

### Dynamic light scattering

The hydrodynamic radius of the liposomes was determined by dynamic light scattering (Nanosizer Nano ZS, Malvern Instruments, UK) in deionized water. Samples were equilibrated for 180 s before 3 × 30 runs (10 s/run) were carried out at 25°C (laser wavelength, λ = 633 nm). Scattering counts were detected at an angle of 173°, and intensity‐weighted sizes and apparent distributions were obtained from the standard cumulant analysis.

### Analytical ultracentrifugation

Analytical ultracentrifugation was performed as previously described (Cinar *et al*, 2020), using an Optima AUC analytical ultracentrifuge (Beckman Coulter, USA) with an An‐50 Ti eight‐hole rotor double‐sector epon centerpieces with a 12 mm optical solution path length. The cells were filled with 420 μl of liposomes diluted in histidine buffer and with 440 μl of buffer in the reference sector. For analysis of the sedimentation velocity data, the ls‐g*(s) model in Sedfit (version 15.01b) was used with confidence levels (*F*‐ratios) set to 0.68 (Schuck & Rossmanith, 2000). This model represents a least‐squares boundary analysis under the assumption of non‐diffusing species and resolves the apparent differential distribution of sedimentation coefficients, allowing further conclusions on the samples (Fig 1).

### Cryo‐transmission electron microscopy

Cryo‐transmission electron microscopy (Cryo‐TEM) was performed on an FEI Tecnai G² 20 system (Thermo Fisher Scientific, USA) at an acceleration voltage of 200 kV. Samples were vitrified utilizing a Vitrobot Mark IV system. Samples were blotted (8.5 µl) on Quantifoil grids (R2/2) and plunge‐frozen in liquid ethane (blot force equivalent −3 mm and a blotting time of 1 s). The diameters were analyzed from a minimum of 500 liposomes per group using FIJI. Geometric size (diameter) distributions were plotted as violin/boxplot using R GNU (Tierney, 2012).

### Ethical statement

All experimental procedures on animals were approved by the local government authority of Thuringia, Thüringer Landesverwaltungsamt, after evaluation and recommendation by an independent ethical advisory board, and carried out following the approved guidelines. FVB/N mice were housed under specific pathogen‐free conditions in the animal facility of the Jena University Hospital. All experiments were performed on mixed populations of male and female mice. For sepsis experiments, the researchers inducing the initial insult were blinded to the distribution of the mice into the different treatment groups. An informed consent was obtained from all subjects via the local ethic committee of the Jena University Hospital on 27 May 2019 (No. 20190527), which approved the study. The experiments adhere to the principles set out in the WMA Declaration of Helsinki and the Department of Health and Human Services Belmont Report. Primary human hepatocytes are obtained from Lonza (Switzerland). Established ethical practices of the donation and transplantation organizations in the US (American Association of Tissue Banks, Association of Organ Procurement Organizations, Eye Bank Association of America) are followed at Lonza. Informed consent, legal authorization and protection of human subject considerations are followed during all steps of the tissue acquisition process. Protected health information is maintained confidentially to protect the privacy of donors as intended by Health Insurance Portability and Accountability Act of 1996 regulation. Lonza holds a permit to operate a tissue bank in the state of Maryland and a tissue bank license from the state of New York for non‐transplant anatomic tissue.

### Polymorphonuclear leukocyte isolation

A crude polymorphonuclear leukocyte (LEU) preparation was isolated from 10 ml human blood of healthy volunteers (heparinized) as previously described by Kuhns *et al* (2015) In brief; the cell fraction was first separated from platelet‐rich plasma by centrifugation (400 *g*, 10 min, ambient temperature). The platelet‐rich plasma was centrifuged again at 2,000 *g*, 10 min, and at ambient temperature to obtain the autologous serum used in a later step. The cell layer was transferred into a 50 ml conical tube, which was filled with HBSS (no calcium, no magnesium) to a volume of 25 ml. 25 ml 3% dextran in HBSS was added, and the closed tube was then inverted five times, and cells were allowed to sediment for approximately 35 min. The RBC‐free supernatant containing the leukocytes was transferred into a fresh tube, filled to 50 ml with HBSS, and the leukocytes spun down at 400 *g* for 10 min at ambient temperature. The cell pellet was then resuspended in ice‐cold 1× RBC Lysis Buffer (420301, Biolegend) and left on ice for 5 min before cells were spun down. The leukocyte pellet was taken up in 2 ml HBSS and overlaid to a discontinuous Percoll gradient (Bottom layer: 2 ml of 51% (v/v) Percoll, Middle layer: 2 ml 42% (v/v) Percoll, both prepared from Percoll (P4937, Sigma‐Aldrich) solution and autologous plasma). The isolation of polymorphonuclear leukocytes (LEU) was realized by centrifugation at 400 *g* for 10 min and by retrieving the LEU from the bottom in 2 ml HBSS and followed by washing once and centrifugation. The pellet was used for further analysis.

### Hepatocyte isolation, cultivation, and stimulation

Primary murine hepatocytes were isolated from male and female FVB/N mice. Animals had been sacrificed by overdose with ketamine and xylazine. All procedures were carried out after the death of the animal was confirmed. The abdomen was disinfected, opened, and the liver perfused through the portal vein with various buffers prewarmed at 40°C (roughly 38°C when reaching the liver tissue) at 8 ml/min. First Krebs‐Henseleit Buffer (Biochrom, Germany) containing 2 g/l glucose and 4 U heparin per ml was perfused for 5 min, followed by perfusion with Hepatocyte Digest Medium (HDM, Thermo Fisher Scientific) for 10–15 min until the tissue was visually digested. Hepatocytes were strained through a mesh (70 µm, Corning) into 50 ml conical tubes and purified by centrifugation at 40 rcf, 4 min, 4°C three times. The hepatocytes in the pellet were kept and supernatant carefully replaced with Hepatocyte Wash Medium (Thermo Fisher Scientific) in between centrifugation steps. Primary human hepatocytes (huHEP; Lonza, Switzerland) and isolated murine hepatocytes (muHEP) were seeded on rat‐tail collagen (10 µg/cm, Merck Millipore) coated well plates at a density of 0.1 × 10^6^ cells per cm². HuHEP were seeded in supplemented hepatocyte plating medium (HPM; #MP100, Lonza, Switzerland) at 37°C. After 1 h, the medium was carefully replaced with supplemented hepatocyte maintenance medium (HMM; #MP250, Lonza, Switzerland) until stimulations. MuHEP were seeded and cultivated in Williams E Medium (PanBiotech, Germany) supplemented with stable glutamine (GlutaMaxx, Thermo Fisher Scientific), insulin (Sigma Aldrich, Germany), and hydrocortisone (Sigma Aldrich, Germany), 10% fetal calf serum (Thermo Fisher Scientific), and Penicillin/Streptomycin (ThermoFisher Scientific; supplemented WEM).

After 16 h, primary hepatocytes were stimulated with a mix of 100 ng/ml LPS (from *Escherichia coli* O111:B4, #L2630, Sigma‐Aldrich), and species autologous cytokines: 10 ng/ml IFN‐γ, 10 ng/ml IL‐1β, and 50 ng/ml TNF‐α (Prospec Biotech, Saudi Arabia) for 24 h in supplemented WEM (muHEP) or HMM (huHEP). Afterward, cells were briefly washed in ice‐cold PBS and lysed in RIPA buffer containing phosphatase inhibitor cocktail (PhosStop, Roche, USA) and protease inhibitor cocktail (HaltProtease Inhibitor Cocktail, Thermo Fisher Scientific, USA).

### Uptake of liposomes in primary human hepatocytes

Transporter‐qualified human hepatocytes from different donors were purchased from Lonza. Hepatocytes were seeded collagen‐coated in 96‐well plates using HPM and HMM as described above. The next day, after an equilibration period of approximately 8 h with fresh HMM, half of the cells were placed on ice for another 60 min. A twofold concentrated ciclosporin A and liposome mixture (pre‐tempered at 37°C or 4°C) was added to the same amount of culture medium to start the experiment. After 30 min, another volume of ice‐cold stop solution (10 mmol/l ciclosporin A) was added, and cells spun down at 1,000 rcf for 3 min. Cells were quickly washed with HBSS by centrifugation and lysed in 10% ethanol in PBS by several freeze‐thaw cycles. Solutions were then cleared by centrifugation at 4,000 *g* for 10 min and the supernatant analyzed in a black 96‐well microtiter plate using an EnSpire (PerkinElmer, USA) microplate reader. Excitation/Emission wavelength of 630 nm/660 nm (with 9 nm band‐passes around the maximum) and a standard in 5% ethanol, PBS is utilized for quantification. 10 µl of the solution was transferred to a 96‐well plate, and 0.2 ml BCA reagent (BCA Macro Kit, Serva Gel Electrophoresis GmbH) was added. The protein concentration was determined using a bovine serum albumin standard curve in 10% ethanol/PBS. Absorbance was measured after 30 min incubation at 37°C at 562 nm. Background (cells not incubated with liposomes) was subtracted for all samples, and dye concentrations normalized to the protein amount in each individual well. Experiments with hepatocytes of different donors were performed. Four individual wells for each condition were analyzed per run. Data were analyzed and plotted using v3.2.0 64× together with the GRmetrics and ggplot2 plugin (Tierney, 2012; Hafner *et al*, 2016).

### Assay of AS605240 activity *in vitro*

The activity of the PI3K inhibitor AS605240 (AbMole BioScience, USA) was assessed in primary human hepatocytes (Lonza, Switzerland) cultivated in collagen type I (10 µg/cm) well plates for 16 h prior stimulation. The next day, cells were pretreated with 200 nmol/l AS605240 in 0.01% DMSO/PBS (w/o calcium and magnesium) or as a liposomal formulation for 1 h in HMM. Controls were pretreated either with either a 0.01% DMSO/PBS‐solution. Cells were then stimulated for 20 min with 1 µmol/l fMLP (Sigma Aldrich) at 37°C, washed with ice‐cold PBS (w/o calcium and magnesium), and lysed immediately in RIPA‐Buffer containing phosphatase inhibitor cocktail (PhosStop, Roche, USA) and protease inhibitor cocktail (HaltProtease Inhibitor Cocktail, Thermo Fisher Scientific, USA). Protein content was quantified using the colorimetric bichinonic acid method (BCA Protein Assay Macro Kit, Serva Electrophoresis GmbH, Germany) and the same amount (25 µg) of protein loaded on an HSE Mini Gel (Serva Electrophoresis GmbH, Germany). Proteins were then transferred to 0.45 µm PVDF membranes (Carl Roth, Germany). Membranes are subsequently incubated in IgG‐free 5% bovine serum albumin (BSA, CarlRoth, Germany) in tris‐buffered saline containing 0.1% (v/v) Tween‐20 (Carl Roth, Germany; TTBS) for 1 h at ambient and stained using a Phospho‐Akt (Ser473; D9E) and Histone H3 (D1H2; all Cell Signaling Technologies, UK) diluted 1:1,000 in 5% BSA in TTBS. Membranes were developed after incubation with cross‐adsorbed HRP‐conjugated anti‐rabbit antibody (#7047, Cell Signaling Technology) diluted 1:2,000 in 5% BSA using ServaLight Eos CL or EosUltra CL HRP Substrate (Serva Electrophoresis GmbH, Germany) and a LAS ImageQuant 4000 (GE Healthcare, USA). Membranes were then stripped for 10 min in 200 mmol/l glycine, 1% (m/v) SDS and 1% Tween‐20 at pH 2.2, washed in PBS and TTBS (two times circa 10 min per buffer), blocked as described above and reprobed using a pan anti‐Akt (C67E7; #4691, Cell Signaling Technology) antibody (diluted 1:1,000 in 5% BSA in TTBS) with the same detection system as described above.

The pAKT/AKT ratio was assessed by Western blot as an indicator for the activity of the PI3Kγ and effects of the (liposomal) PI3Kγ inhibitor AS605240. Experiments were performed with hepatocytes from three different donors. The blots were analyzed densitometrically using ImageJ distribution Fiji v1.53g 64× Gel‐analyzer plugin (Schindelin *et al*, 2012; Schneider *et al*, 2012).

### Detection of PI3Kγ in cell lysates

Purified human hepatocytes from 20 male or 20 female donors (HEP), an isolated non‐parenchymal cell fraction (NPC) pooled from three male donors containing cholangiocytes, Kupffer cells, and liver sinusoidal endothelial cells, and the purified crude polymorphonuclear leukocyte (LEU) fraction were lysed in RIPA buffer containing protease and phosphatase inhibitor (3 × 10^6^ cells/ml). HEP and NPC were purchased from Lonza, Switzerland. 5 µl of each lysate (HEP and LEU) and 15 µl NPC were used for the detection of PI3Kγ by Western blot. Alternatively, approximately 20 µg of protein from cultivated primary human or murine hepatocytes lysates were used.

The Western blot was performed as described above and developed using ServaLight EoS CL Luminol and the LAS ImageQuant 4000 detection system (GE Healthcare, USA). The human PIK3CG (PI3Kγ; #NBO2‐15071, Novus Biologicals) was diluted 1:2,000 in 5% BSA in TTBS and incubated overnight at 4°C, rocking with the membrane.

### Immunohistochemistry

Biopsies from patients with various mild to severe liver diseases (Appendix Table S2) were investigated. Paraffin‐embedded liver tissue sections (4 µm thickness) mounted on glass slides are used for the following staining procedure. Tissue sections were deparaffinized and permeabilized using fresh citric acid buffer (10 mmol/l sodium citrate, 0.05% Tween 20, adjusted to pH 6.0 with 1 mol/l HCl) for 25 min. Slides rinsed with TTBS were blocked for 10 min at room temperature using DAKO peroxidase blocking solution (#S200230, Dako), washed again in TTBS, blocked in donkey serum (Equitech‐Bio, #SD30‐0100), for 1 h at room temperature, and incubated in primary antibody, rabbit polyclonal anti‐PIK3CG (PI3Kγ; NBP2‐15071, Novus Biologicals) at 4°C overnight diluted 1:200 in antibody diluent (#S3022, Dako). Negative controls were incubated with antibody diluent only. The next day the slide were rinsed in TTBS, incubated in HRP‐conjugated goat anti‐rabbit IgG (ab214880, Abcam) diluted 1:200 in antibody diluent for 2 h at room temperature, washed in TTBS, and incubated in DAB Substrate solution (SignalStain DAB Substrat Kit, #8059, Cell Signaling Technology). After 10 min, slides were carefully rinsed with deionized water and counterstained with hematoxylin. Washed and ethanol‐fixed tissue sections were then mounted using Eukit (#03989, Sigma Aldrich). Images were acquired on an Olympus AX70 inverted microscope equipped with an AxioCam MRc5 (operated using AxioVision SE64 Rel. 4.9), a Halogen Lamp (U‐PS, Olympus), a 20×/0.5, and 40×/0.75 semi‐apochromatic objectives (Olympus).

### Animals, survival, and tissue sampling

PI3Kγ null (knockout) (Hirsch *et al*, 2000) and liver‐specific PI3Kγ knockout mice (PI3Kγ flox^tg/tg^ × AlbCre^(tg)/tg^) (Postic *et al*, 1999), as well as the background strain C57BL/6J were bred in the animal facility of the Jena University Hospital. Pharmaceutical inhibition of the PI3Kγ by AS605240 or liposomal formulations was carried out in FVB/N mice (8–12 weeks). The Jena University Hospital's animal facility provided a specific pathogen‐free environment for all experiments and standardized husbandry conditions (50–60% humidity, a 12‐h light/dark cycle, and a constant ambient temperature (24°C), free access to standard rodent chow and drinking water).

All animals were either subjected to peritoneal contamination and sepsis by injecting a characterized human stool suspension (PCI group) (Gonnert *et al*, 2011; Sommerfeld *et al*, 2020) or saline (sham group) intraperitoneally. Our studies were designed to fulfill the minimum quality threshold in pre‐clinical sepsis studies, including antibiotic and analgesic treatment as well as fluid resuscitation (Osuchowski *et al*, 2018). The dose of human stool suspension injected intraperitoneally was optimized for the C57BL/6J and FVB/N background aiming at a 7 day‐survival of 50% mortality (C57BL/6J) or 75% (FVB/N) in wild‐type or untreated mice, respectively. At 6 h after infection, antibiotics and fluid resuscitation (meropenem 2.5 mg per kg body weight dissolved in Ringer's solution to a volume of 2.5 mg/ml) were administered every 12 h subcutaneously for 7 days. Metamizole (oral, all experiments on FVB/N mice) or buprenorphine (subcutaneous, experiments on C57BL/6J and genetically engineered mice) was given for pain relief. A treatment‐free interval between the infection and first treatment was introduced to mimic the clinical situation best. DMSO‐dissolved AS605240 (AS; 4 mg/kg body weight) or DMSO (Vehicle, Veh; 4 mg/kg body weight) solution was administered intraperitoneally. T‐LipoAS, T‐Lipo, LipoAS, or Lipo were injected intravenously (injected liposomes 0.1 mg/kg body weight). Treatment with the different inhibitors was applied for the first 5 days beginning at 6 h after infection and repeated every 24 h. Under isoflurane anesthesia, organs were harvested for analysis after 24 or 48 h. At the time‐point of analysis (24 h), animals may fully develop sepsis‐associated liver failure, but at the same time, no mortality is detected, making the earliest chosen time‐point unbiased concerning the selection of potential survivors and non‐survivors. The death occurred following a terminal cardiac puncture to collect EDTA plasma. Post‐mortem lavage of the peritoneum was performed with 2 ml PBS containing 5 mmol/l EDTA.

### Neutrophil infiltration

Neutrophil infiltration in the peritoneal cavity was assessed by flow cytometry. Cells from 0.5 ml lavage were concentrated by centrifugation (1,000 *g*, 10 min) and stained against CD45, CD11b, CD115, and Ly6‐C (Watson *et al*, 2015). Identified neutrophil populations (CD45^pos^., CD11b^pos^., CD115^neg^., Ly6‐C^low/neg^.) were counted using a BD Accuri (BD Bioscience, USA) and flow software (v2, Perttu Terho, University of Turku, Finland). The gating strategy is depicted in Appendix Fig S6.

### Plasma disappearance rate

FVB/N were anesthetized with isoflurane and warmed during the experiment. The hair on the ear was removed with commercial hair removal cream (Silk’n’fresh, Veet). The ear was immobilized on a cover slide mounted in an LSM‐780 (Zeiss AG, Jena, Germany). The LSM‐780 was operated in a pseudo‐widefield mode (open pinhole) to minimize artifacts from eventual drifts in Z‐direction. Ear veins were identified as negative contrast utilizing the surrounding tissue's autofluorescence. Images were taken every 30 s from multiple positions and veins of each animal over 45 min. T‐LipoAS (50 µg) was injected in histidine solution utilizing a tail‐vein catheter 1 min into the imaging. A 633 nm Helium‐Neon laser excited the targeting unit (DY‐635), and fluorescence was recorded through a band‐pass (640–780 nm) with a Plan‐Apochromat 20× 0.8 objective (Zeiss AG, Jena, Germany). The DY‐635 mean fluorescence intensity originating from veins after T‐LipoAS injection was collected employing the FIJI (v 1.53j) distribution of ImageJ for regions of interest (ROIs) placed in all veins recorded. Then, the autofluorescence from the first image was subtracted from the stack. The linear regression in the constant decay phase of all curves (15–40 min) resulted in individual half‐lives (for each vein) that were then averaged. A total of 32 regions from 4 male FVB/N mice (8 weeks old) had been analyzed. Eight regions were excluded due to no initial signal increase or apparent Z‐drift. The mean fluorescence intensity averaged between the remaining 24 regions of interest (from *n* = 4) and the positive standard error depicted in the figure.

### Liver intravital microscopy

FVB/N mice with (PCI) and without (sham) sepsis were treated with (nanoformulated) inhibitors or vehicle solutions plus antibiotics and metamizole analgesia, as described above. Intravital microscopy was performed at 24 h post‐infection. 1 h before surgery, carprofen (5 mg/kg body weight, s.c.) was administered. Surgery and subsequent intravital microscopy were carried out under deep isoflurane anesthesia. Animals were kept warm using a heating pad or on a warmed (34°C), humidified microscopic chamber. The surgery required to perform intravital microscopy has been previously described (Press *et al*, 2017). Liposomes and dyes were administered through a tail‐vein catheter. DY‐635 conjugated liposomes were administered to some animals. To investigate biodistribution during sepsis, CDFDA was injected to probe hepatocyte function. At the end of the experiment, propidium iodide (Thermo Fisher Scientific, USA) and Hoechst 33342 (Sigma Aldrich, Germany) were injected for live/dead cell discrimination. Animals were sacrificed at the end of the experiment while remaining under anesthesia.

### Multispectral optoacoustic tomography

Animals were anesthetized and placed in the Multispectral Optoacoustic Tomography inVision 256‐TF (iTheraMedical, Germany). A liver section was then focused in the tomograph. An optoacoustic dataset was acquired every 15 s for the duration of 20 min. Ten images per excitation wavelength were averaged from two different excitation wavelengths at each time point, 800 and 900 nm for ICG and mouse anatomy, respectively. A baseline of 2 min was acquired before injecting approximately 20 µg indocyanine green, ICG (Verdye, Diagnostic Green, USA) intravenously through a tail‐vein catheter. The acquired images were pre‐processed using software ViewMSOT, iTheraMedical GmbH (Release 3.8.1.04). The images were reconstructed by back‐projection mode with a filter range of 50 kHz to 6.5 MHz‐IR. After reconstruction, image analysis was carried out on the processed multispectral MSOT hyperstacks by using ImageJ distribution Fiji v1.49b 64× (Schindelin *et al*, 2012; Schneider *et al*, 2012) and R v3.5.0 64× (Tierney, 2012). The time‐resolved, multispectral MSOT hyperstacks were first subdivided into two image stacks: one representing the signal from anatomical structures obtained by illumination with 900 nm laser wavelength, the other corresponding to the ICG signal obtained by illumination with 800 nm laser wavelength. Regions of interest (ROIs) were drawn manually around animal contours in the first cross‐sectional time frame guided by the anatomical image stack. In order to compensate for motion artifacts, rigid image registration was performed using the Fiji plugin MultiStackReg v1.45 (Busse, Bard Science Downloads), which is based on a pyramid approach to subpixel intensity‐based registration (Thévenaz *et al*, 1998). To this end, the anatomical image stack was used to obtain transformation matrices for each time frame, which were then applied to the corresponding time frames of the ICG image stack. To adjust for intrinsic intensity variations between scans and to ensure inter‐scan comparability, intensity values of the ICG image stack were z‐transformed according to

(1) $$Z_{x,y,z}=\frac{X_{x,y,z}‐\mu }{\sigma },$$

where *Z_x_*_,_ *_y_*_,_ *_z_* is the *z*‐transformed intensity value at pixel position *x*, *y* in time frame *z*, *X_x_*_,_ *_y_*_,_ *_z_* is the original intensity value at pixel position *x*, *y* in time frame *z* and *μ* and σ are the mean and standard deviation of intensity values of the first‐time frame. The time‐resolved ICG image stack was then down sampled by 4 in the time domain to reduce the impact of animal breathing and was cropped to the bounding box of the ROI. All pixel values outside the ROI were set to zero. For each pixel in every ICG image stack, a vector of the signal intensity change rates between consecutive time frames was exported for further analysis. *K*‐means clustering (*k* = 4) was applied to extract main characteristic time curves of signal intensity change rates for each treatment group (sham vehicle, sham T‐LipoAS, PCI vehicle, and PCI T‐LipoAS). For each animal in a particular group, all pixel positions were compared with the characteristic curves that were obtained by *k*‐means clustering for this treatment group. Each pixel position was assigned to belong to the characteristic curve that had the smallest Euclidean distance to the vector of signal intensity change rates at this pixel position. In that way, pixel abundance values were obtained for each animal with regard to the characteristic curves of the corresponding treatment group. To restrict the analysis to ICG uptake, only characteristic curves that represent a net increase of the ICG signal intensity over time were used for further quantification. The weighted average of those curves was calculated for each animal, using the pixel abundance values as weights and dividing by the sum of the respective pixel abundances. Tailing the linear part of the thus resulting curves was then identified by visual inspection and found to start at time frame 8 (down‐sampled time domain). Finally, the area under the curve was calculated for this linear part for each animal and used to compare treatment groups.

### Episcopic imaging of biodistribution

Mice injected with T‐LipoAS were sacrificed and frozen post‐mortem in TissueTek (OCT Compound, USA). An episcopic setup was applied on a cryomicrotome (CM3050 S Cryostat, Leica, Germany) for imaging of the whole mouse. DY‐635 was excited with an LED at 625 nm wavelength (M625L4, Thorlabs, USA) and additionally filtered by a 1″ band‐pass filter with a center wavelength of 630 nm and FWHM of 20 nm (ET630/20x, Chroma, USA). Fluorescence was observed through a 2″ band‐pass filter with a center wavelength of 670 nm and FWHM of 30 nm (ET670/30m, Chroma, USA). The sample was observed by a monochrome camera with 4,500 × 3,600 pixels and 6 µm pixel size (MicroLine ML16200, FLI Instruments, USA) equipped with a 1:1 lens with 89 mm focal length using f/4.8 (XENON‐ZIRCONIA 2.8/89, Schneider Kreuznach, Germany). RGB images were generated by illuminating the sample sequentially with a ring of red, green, and blue LEDs (24× SK6812RGBW‐WS, Opsco, China).

### Plasma cytokine analysis

The Mouse Inflammation Panel 13‐plex (LegendPlex, Biolegend, USA) was used to measure cytokine levels in EDTA plasma using filter plates. The samples were prepared, measured, and analyzed using the BD Accuri according to the manufacturer's instructions.

### Electron microscopy

For electron microscopy of the liver, animals were sacrificed at 24 h after the septic insult. The abdomen was opened and the liver perfused through the portal vein with buffer followed by a glutaraldehyde and formaldehyde based‐fixative as described previously (Schaarschmidt *et al*, 2018). Samples were washed with Dulbecco's phosphate‐buffered saline (DPBS, Sigma Aldrich, Germany) and subsequently processed by osmium tetroxide post‐fixation (1% OsO_4_ in DPBS for 2 h), and dehydration in a graded series of increasing ethanol:water mixtures (30, 50, 70, 90, 96 and 100%) for at least 10 min each. Embedding was performed by applying EPON812 embedding solutions (1:2 Epon: ethanol for 1 h, 1:1 for 2 h, and 100% Epon overnight followed by a final exchange with 100% Epon and DMP). Samples were cured for 24 h at 60°C. Samples were subsequently trimmed and sliced into 100–150 nm thick samples utilizing a PTPC Ultramicrotome (RMC Boeckeler, Germany) facilitated with a diamond knife (Diatome, Germany). Slices were placed on formvar/carbon‐coated nickel finder grids (Plano, 200mesh). For improving the imaging contrast, samples were stained with 2% uranyl acetate solutions for 30 min. Subsequently, all samples were analyzed by transmission electron microscopy (FEI Tecnai G² 20 at an acceleration voltage of 200 kV). Images were recorded utilizing an OSIS (Olympus Soft Imaging Solutions, USA) CCD camera with 1,376 × 1,024 pixels and analyzed by ImageJ software.

### Statistics

Data processing and statistical testing were done in R GNU (v. 3.1 or higher) using the RStudio interpreter. Non‐parametric tests had been performed when normality or equal variances could not be assumed. Data points in the graphs depict individual replicates if not explicitly mentioned otherwise. Detailed information on statistical tests, replicates, and significance levels are provided in the figure legends and additional considerations in the “Appendix Tables”.

## Conflict of interest

The authors declare that they have no conflict of interest.

## Author contributions

Animal and cell culture experiments, imaging, and tissue analysis: ATP, PB, and JB; Characterization of nanomaterials: ATP, WF, IN, SH, and TM; Cell culture experiments: MaBe; Algorithms and MSOT data analysis: BH and ZC; Development, performance, and analysis of episcopic imaging WH, WF, and ATP; Supervision of nanomaterial characterization: SMC and MHG; Supervision of experiments on human tissue sections: NG; Performance of animal studies: RB and FG; Study guidance: RW; Supervision of the materials science concept: USS; Supervision and guidance of MSOT image analysis: MTF; Project design and guidance: ATP and MiBa; Writing the manuscript: All authors.

## For more information

https://www.uniklinikum‐jena.de/cscc/en/

https://www.sepsis.org/

https://www.who.int/health‐topics/sepsis#tab=tab_1

## Supporting information



Appendix

## Data Availability

All data required for the evaluation of the study are presented in the manuscript and deposited on a server of the Hans‐Knoell Institute Jena (https://asbdata.hki‐jena.de/PressEtAl2021_EMBOMolMed).
