# Supplementary material for: Targeted delivery of a phosphoinositide 3‐kinase γ inhibitor to restore organ function in sepsis
Source: EMBO Mol Med. 2021 Sep 2;13(10):e14436. doi: 10.15252/emmm.202114436 (PMC8495460; doi:10.15252/emmm.202114436)
Supplement: Supplementary file 1 — Appendix [file EMMM-13-e14436-s002.pdf]

APPENDIX

Targeted delivery of a phosphoinositide 3-kinase γ inhibitor to restore organ function in sepsis through dye-functionalized lipid nanocarriers

Authors Adrian T. Press[1,2,3], Petra Babic[1,3], Bianca Hoffmann[4], Tina Müller[1,3], Wanling Foo[1], Walter Hauswald[5], Jovana Benecke[1,3], Martina Beretta[1,3], Zoltán Cseresnyés[4], Stephanie Hoeppener[6,7], Ivo Nischang[6,7], Sina M. Coldewey[1,3,8], Markus H. Gräler[1,3], Reinhard Bauer[10], Falk Gonnert[1,3], Nikolaus Gaßler[9], Reinhard Wetzker[1,3], Marc Thilo Figge[2,4,11], Ulrich S. Schubert[3,6,7], Michael Bauer[1,3,6]

Affiliations

[1] Department of Anesthesiology and Intensive Care Medicine, Jena University Hospital, Am Klinikum 1, 07747 Jena, Germany

[2] Medical Faculty, Friedrich Schiller University Jena, Bachstr. 18, Jena, Germany

[3] Center for Sepsis Control and Care, Jena University Hospital, Am Klinikum 1, 07747 Jena, Germany

[4] Research Group Applied Systems Biology, Leibniz Institute for Natural Product Research and Infection Biology - Hans Knoell Institute, Jena, Germany

[5] Leibniz Institute of Photonic Technology, Albert-Einstein-Straße 9, 07745 Jena, Germany

[6] Jena Center for Soft Matter (JCSM), Friedrich Schiller University Jena, Philosophenweg 7, 07743 Jena, Germany

[7] Laboratory of Organic and Macromolecular Chemistry (IOMC), Friedrich Schiller University Jena, Humboldtstrasse 10, 07743 Jena, Germany

[8] Septomics Research Centre, Jena University Hospital, Jena, Germany

[9] Institute of Molecular Cell Biology, Jena University Hospital, Jena, Germany

[10] Section of Pathology, Institute of Forensic Medicine, Jena University Hospital, Am Klinikum 1, 07747 Jena, Germany

[11] Institute of Microbiology, Faculty of Biological Sciences, Friedrich Schiller University, Jena, Germany

Correspondence to *michael.bauer@med.uni-jena.de*

Table of Content

Appendix Figure S 1: Cryo-Transmission electron microscopy with size quantification of non-targeted Liposomal formulation. 3

Appendix Figure S2: Analytical ultracentrifugation of AS605240 loaded liposomes......... 4

Appendix Figure S3: Effects of targeted AS605240 delivery on the inflammatory response. 5

Appendix Figure S4: Scheme on processing and analysis of multispectral optoacoustic tomography (MSOT) images for the quantification of liver function by indocyanine green (ICG) clearance. 6

Appendix Figure S5: Effects of the non-targeted liposomal AS605240 formulation on survival in the peritoneal contamination and infection (PCI) sepsis model. 7

Appendix Table S1: PI3Kγ expression in human tissue biopsies. 9

Appendix Table S2: Characterization of primary human hepatocyte donor pools (Lonza, Switzerland)... 10

Appendix Table S3: p-Value Tables for Figures 2 to 5 in the main manuscript and Appendix Figure S3 and S5.. 11

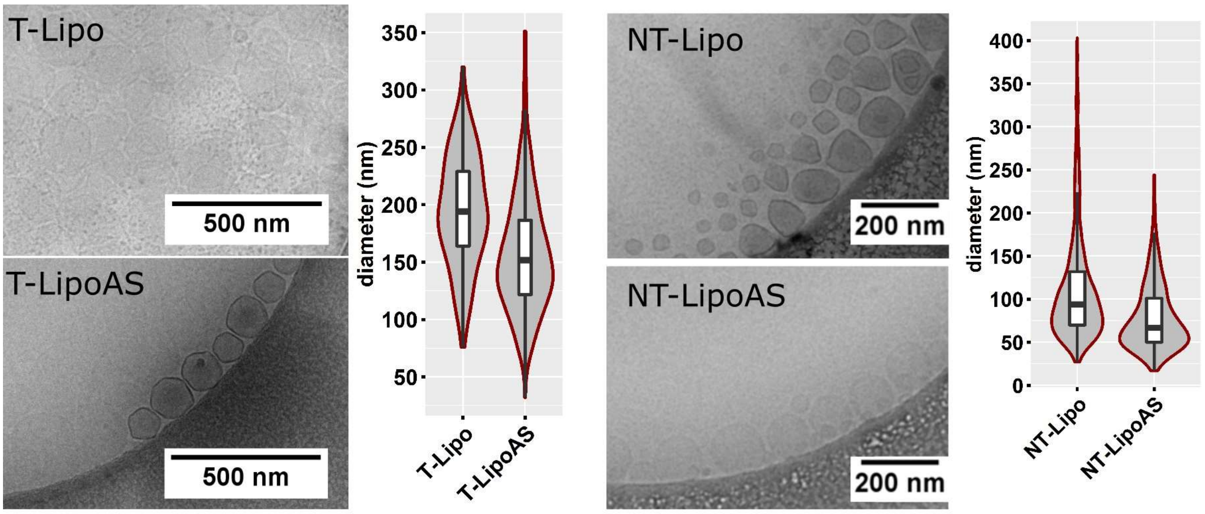

Appendix Figure S 1: Cryo-Transmission electron microscopy with size quantification of non-targeted Liposomal formulation.

Diameter as measured from **TEM images** (ImageJ). Median box-plot with 0.25;0.75 IQR; whiskers 0.95 percentile, violin-plots depict density. NT-Lipo 14 images = 847 liposomes, NT-LipoAS 15 images = 540 liposomes, T-Lipo 5 images = 184 liposomes, T-LipoAS 12 images = 503 liposomes

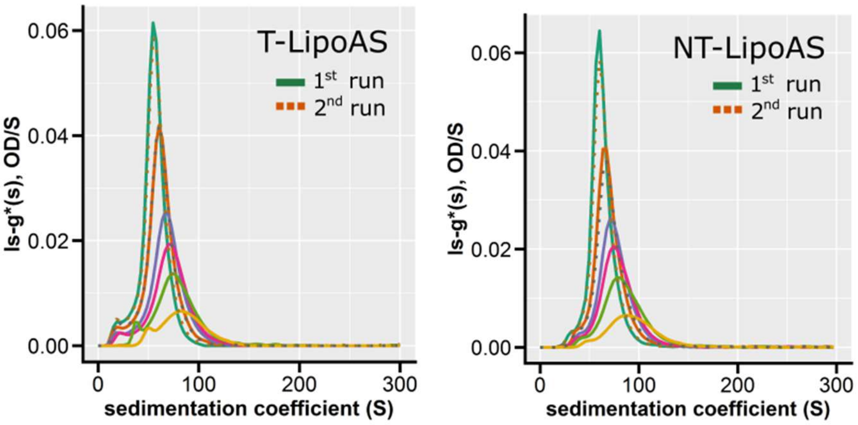

Appendix Figure S2: Analytical ultracentrifugation of AS605240 loaded liposomes.
Analytical ultracentrifugation was performed with different concentrations of liposomes. The optical density monitored the sedimentation at 496 nm. Data analysis was accomplished with the $ls - g^{*}(s)$ model in Sedfit (version 15.01b). The $ls - g^{*}(s)$ model represents a least-squares boundary analysis under the assumption of non-diffusing species and resolves the apparent differential distribution of sedimentation coefficients.

The decrease of liposomal concentration shifted the sedimentation coefficient distributions toward larger values (solid lines). Finally, sedimented liposomes were resuspended by shaking the ultracentrifuge cells, then another sedimentation velocity analysis (dotted lines). Again, very similar results were observed at all concentrations, indicating the stability of liposomes during sedimentation and after resuspension leading to the observation of identical populations.

Each liposomal formulation was analyzed at six different concentrations. The spun down liposomes were resuspended by shaking the cells and re-analyzed, leading to an identical result, indicating stability. Experiments were performed for six different liposome concentrations per formulation in one replicate for each liposomal formulation.

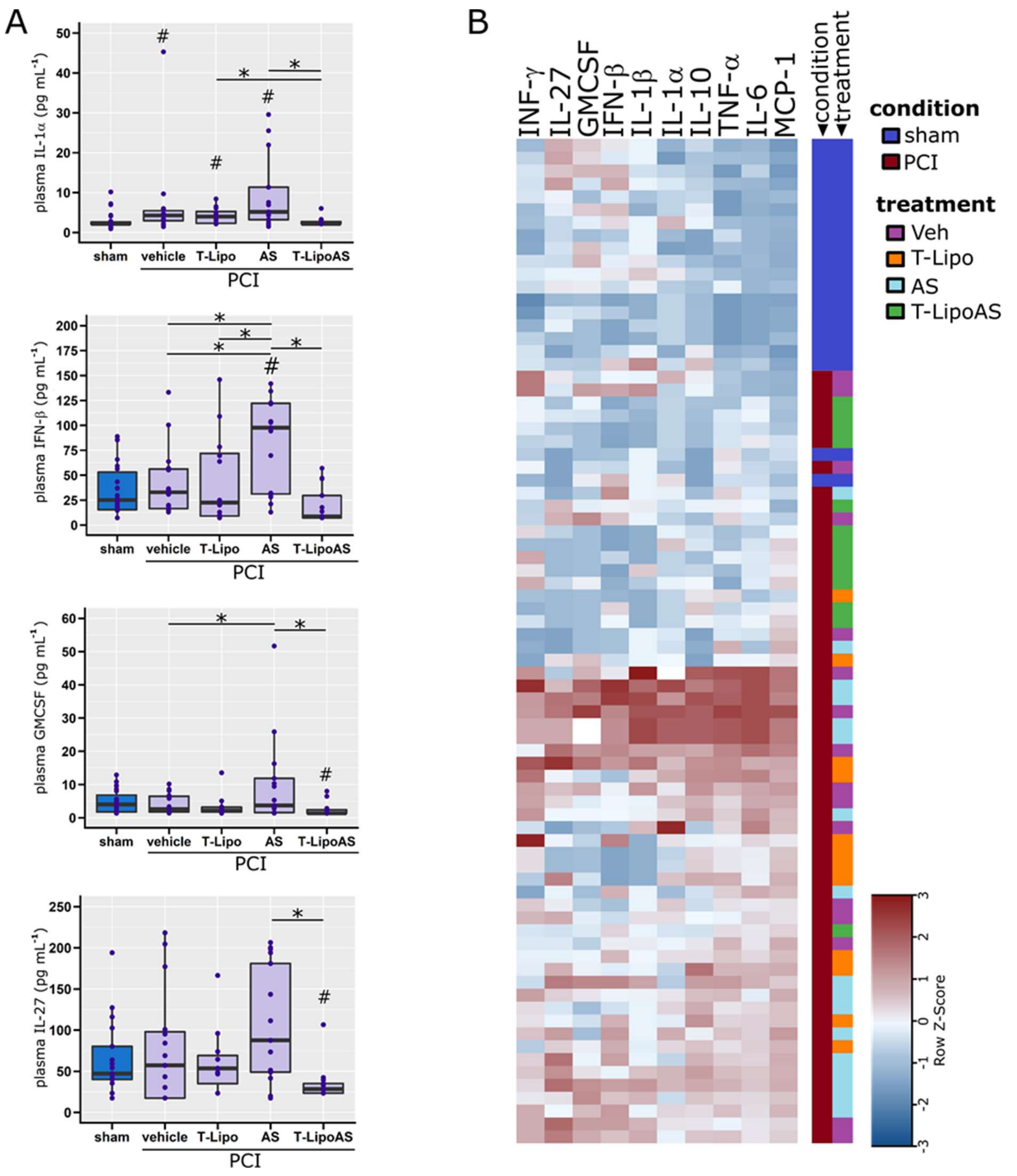

Appendix Figure S3: Effects of targeted AS605240 delivery on the inflammatory response.

(A) Pro-inflammatory-cytokines essential for the host defence were analyzed in EDTA-plasma from PCI and sham animals. #p<0.05 against sham, *p<0.05 as indicated; Kruskal-Wallis ANOVA with controlled false-discovery rates (Benjamini-Hochberg procedure). The p-values are given in Appendix Table S3. **(B)** Cytokine profiles are depicted in a self-organizing heat-map according to their similarity of appearance in plasma. Information on replicates can be found in Figures 3 B and 3 C).

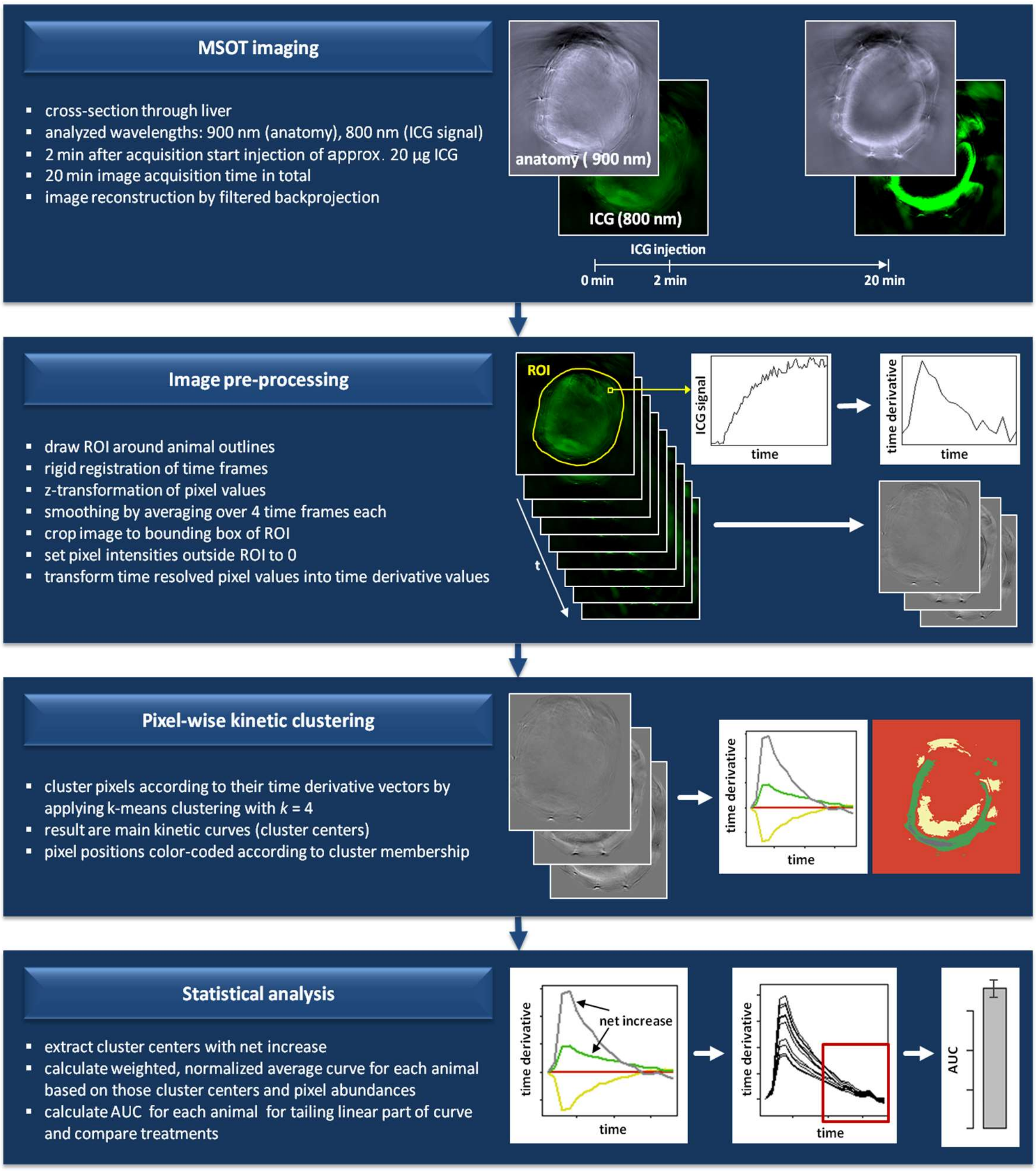

Appendix Figure S4: Scheme on processing and analysis of multispectral optoacoustic tomography (MSOT) images for the quantification of liver function by indocyanine green (ICG) clearance.

MSOT imaging is performed to derive time-resolved cross-sectional images of liver tissue and ICG distribution. MSOT image stacks are first pre-processed to normalize ICG intensity values and smooth time course data. The time derivatives of pixel intensities within a region of interest (ROI) from one-time frame to the next serve as input for pixel-wise kinetic clustering. By applying k-means clustering with k set to 4, 4 main kinetic curves (cluster centers) present in the ROI are extracted, and color-coded images are generated to visualize the spatial distribution of those clusters for each animal. For quantification of ICG signal, only those cluster centers reflecting signal net increase are taken into account and combined into one curve, incorporating the respective abundances of pixels for each animal individually. All known three phases of ICG pharmacokinetics are clearly evident in those curves: first-pass (peak), re-distribution (exponential decay) and the final linear phase representing the dye-clearance through the liver. The area under the curve (AUC) was calculated from the linear decay phase to estimate the elimination capacity of the liver.

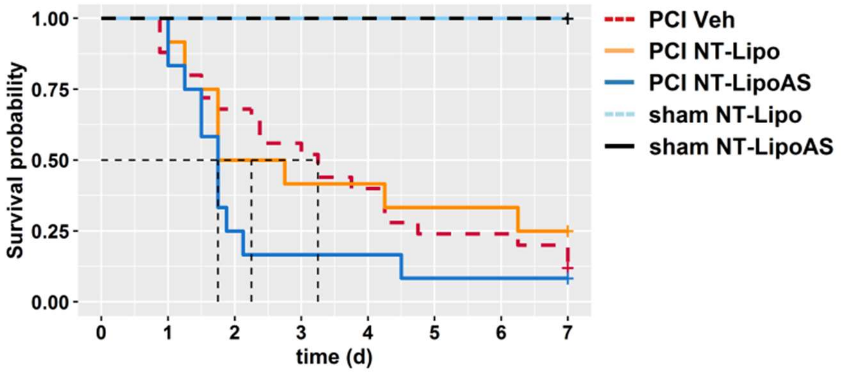

Appendix Figure S5: Effects of the non-targeted liposomal AS605240 formulation on survival in the peritoneal contamination and infection (PCI) sepsis model.

Group sizes for sham groups NT-Lipo: 6 (50% female), NT-LipoAS: 2 (50% female) animals. For PCI (sepsis) groups: Veh: 25 (56% female), NT-Lipo: 12 (50% female), NT-LipoAS: 12 (50% female) animals.The survival is monitored for seven days in this model. In the first two or three days, the acute phase had passed and increasingly processes related to a cornification occur. Animals appear phenotypically healthy after five to seven days. However, it is common in this model for abscesses to occur without visible symptoms, which in some cases may even be macroscopically detected as early as 24 h post-infection, due to the lack of a surgical intervention cleaning and disinfecting the primary site of infection (here: abdominal cavity). These abscesses may grow and eventually rupture, causing spontaneous death unrelated to the actual experiment. As a consequence, the survival is limited here to seven days. The p-values of the statistical analysis are provided in Appendix Table S3.

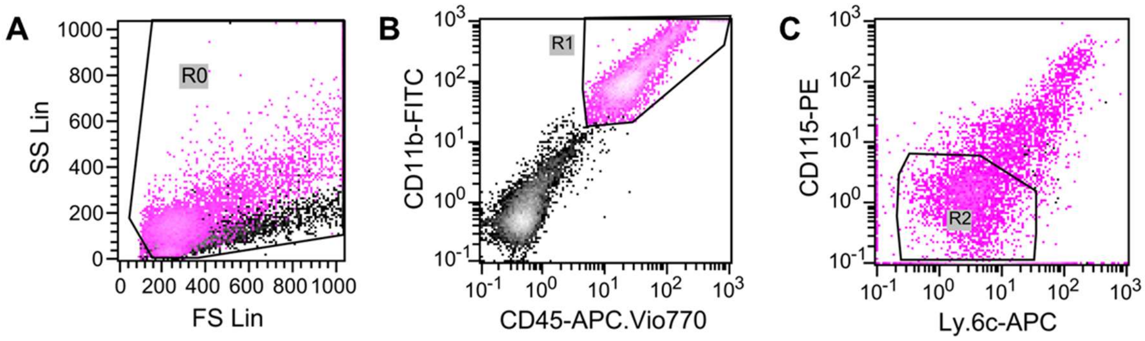

Appendix Figure S6: Gating of Lavage cells for analysis.

Gating was performed as previously described by Watson et al. (J Immunol. 2016, 194(6): 2796-2809, PMID: 25681345). Peritoneal lavage and subsequent processing and staining were carried out as described in the method section. The figure depicts the gating strategy. A stained (pink) and unstained (black/grey) sample is overlaid in the XY-Plots: (**A**) Forward scatter vs side scatter plot with gate R0 to separate cell events from debris. (**B**) Fluorescence labeled double-positive CD11b+ CD45+ cells are then gated (R1) from R0. (**C**) CD115- and Ly-6c negative fraction of R1 is depicted, and R2 gate is used to count cells neutrophils. CD115+, Ly6c+ cells (#R1-#R2, with # stands for the number of cells in each gate) are classified as monocytes. Data are normalized to the volume used for measurement, obtaining the concentration of cells per volume lavage.

Appendix Table S1: PI3Kγ expression in human tissue biopsies.

LSECs: liver sinusoidal endothelial cells, KCs: Kupffer cells (local macrophages)

**Sample information**		**N**	**PI3Kγ expression**			
Diagnosis	Gender		Hepatocytes	LSECs, KCs	Infiltrating immune cells	endothelial cells of larger vasculature
Non-alcoholic fatty liver disease (NASH)	female<br>male	3<br>1	yes	no	Yes	yes
Autoimmune hepatitis (AIH)	male<br>female	1<br>1	yes	no	Yes	yes
Liver cirrhosis	female<br>male	2<br>1	yes	no	Yes	yes

Appendix Table S2: Characterization of primary human hepatocyte donor pools (Lonza, Switzerland).

Parameter	huHEP ♂$_{DP20}$	huHEP ♀$_{DP20}$
Number of Donors	20	20
Donor Age Average	36.9	41.1
Donor BMI Average[a]	26.3	29.2
Male Donors	100.0%	0.0%
Female Donors	0.0%	100.0%
Asian Donors	5.0%	0.0%
Afro American Donors	5.0%	10.0%
Caucasian Donors	80%	85.0%
Other Donors	10.0%	5.0%
No Drugs/ Alcohol/ Tobaco	45.0%	60.0%
Illicit Drug use	35.0%	20.0%
Tobacco Use	35.0%	20.0%
Heavy alcohol use[b]	5.0%	5.0%
Serologies CMV	Positive	Positive
Serologies EBV	Positive	Positive
Serologies HBV	Negative	Negative
Serologies HCV	Negative	Negative
Serologies HIV	Negative	Negative
CYP1A2 (pmol/ 10^6 cells/min, 100 µmol L^{-1} Phenacetin)	68.7	81.7
OATP1B1/3 Active Estrone-3-S Uptake (pmol/ 10^6 cells/min, 10 µmol L^{-1} Estrone-3-S)[c]	1.6	1.9
NTCP Active Taurocholate Uptake (pmol/ 10^6 cells/min, 10 µmol L^{-1} Taurocholate)[c]	2.9	3.5

[a] Body mass index (BMI) is derived from body mass (weight) divided by the square of the body height of a person.

[b] Heavy Alcohol use is defined as >2 drinks per day or current history of alcoholism

[c] Transporter Activity is the amount of specific substrate retained on hepatocytes after incubation with 10 µmol L^{-1} substrates at 4°C or at 37°C for 3 min. Active uptake is the fold change between uptake at 37°C versus 4°C. The uptake is expressed in pmol per 10^6 cells per min.

Appendix Table S3: p-Value Tables for Figures 2 to 5 in the main manuscript and Appendix Figure S3 and S5

Figure 2

Figure 2 E	AS605240 quantification	
Tissue	p-value	W
Lung	0,00729	0
Spleen	0,00729	0
Liver	0,2207	5
Kidney	0,01351	0
Fat	0,00729	0
Brain	1	10

Figure 3

Figure 3 B	Interleukin 6 (IL-6)			
p-value table	sham	PCI Vehicle	PCI T-Lipo	PCI AS
PCI Vehicle	2,1E-05			
PCI T-Lipo	5,3E-05	1,0E+00		
PCI AS	9,1E-06	1,3E-01	1,4E-01	
PCI T-LipoAS	5,3E-05	1,4E-01	1,6E-02	2,5E-03

Figure 3 B	Interleukin 1 beta (IL-1β)			
p-value table	sham	PCI Vehicle	PCI T-Lipo	PCI AS
PCI Vehicle	2,31E-02			
PCI T-Lipo	6,16E-01	0,0231		
PCI AS	1,07E-02	0,6162	0,0107	
PCI T-LipoAS	7,80E-02	0,0014	0,4255	0,0014

Figure 3 B	Tumor Necrosis Factor alpha (TNF-α)			
p-value table	sham	PCI Vehicle	PCI T-Lipo	PCI AS
PCI Vehicle	2,40E-05			
PCI T-Lipo	3,70E-05	0,61407		
PCI AS	3,40E-06	0,01025	0,03152	
PCI T-LipoAS	4,15E-03	0,00141	0,00052	0,0001

Figure 3 B	Interferon gamma (IFN-γ)			
p-value table	sham	PCI Vehicle	PCI T-Lipo	PCI AS
PCI Vehicle	0,0140			
PCI T-Lipo	0,0000	1,0000		
PCI AS	0,0017	0,3563	0,3563	
PCI T-LipoAS	0,0140	1,0000	1,0000	0,3563

Figure 3 B	Macrophage chemotactic protein 1 (MCP-1)			
p-value table	sham	PCI Vehicle	PCI T-Lipo	PCI AS
PCI Vehicle	1,70E-05			
PCI T-Lipo	3,00E-05	0,58064		
PCI AS	4,70E-06	0,0492	0,06094	
PCI T-LipoAS	7,20E-05	0,14442	0,01105	0,00033

Figure 3 C	Interleukin 10 (IL-10)			
p-value table	sham	PCI Vehicle	PCI T-Lipo	PCI AS
PCI Vehicle	6,96E-01			
PCI T-Lipo	2,80E-02	0,1107		
PCI AS	7,30E-03	0,0382	0,554	
PCI T-LipoAS	9,39E-01	0,6858	0,0143	0,0073

Figure 3 D	Bacterial Burden	Lavage							
p-value table	PCI_AS_24h	PCI_AS_48h	sham_24h	sham_48h	T-Lipo_24h	T-Lipo_48h	T-LipoAS_24h	T-LipoAS_48h	vehicle_24h
PCI_AS_48h	0,0227								
sham_24h	0,0094	0,0094							
sham_48h	0,0094	0,0094	0,4526						
T-Lipo_24h	0,0157	0,8081	0,0190	0,2350					
T-Lipo_48h	0,0097	0,1584	0,0190	0,2350	0,4526				
T-LipoAS_24h	0,0094	0,6724	0,0094	0,0097	0,8081	0,3833			
T-LipoAS_48h	0,0097	0,0808	0,0190	0,2350	0,2647	0,5464	0,1169		
vehicle_24h	0,0175	0,1286	0,0094	0,0094	0,3360	0,8081	0,2493	0,3360	
vehicle_48h	0,0079	0,0094	0,0094	0,0097	0,0420	0,1324	0,0175	0,6948	0,1853

Figure 3 E	Bacterial Burden	Liver							
p-value table	PCI_AS_24h	PCI_AS_48h	sham_24h	sham_48h	T-Lipo_24h	T-Lipo_48h	T-LipoAS_24h	T-LipoAS_48h	vehicle_24h
PCI_AS_48h	0,0384								
sham_24h	0,0095	0,0095							
sham_48h	0,0103	0,0103	0,0766						
T-Lipo_24h	0,0521	0,9545	0,0103	0,025					
T-Lipo_48h	0,0103	0,0459	0,0103	0,025	0,0459				
T-LipoAS_24h	0,0208	0,6892	0,0095	0,0103	0,5012	0,025			
T-LipoAS_48h	0,0157	0,992	0,0103	0,025	0,25	0,7526	0,1444		
vehicle_24h	0,025	0,7724	0,0095	0,0103	0,6573	0,0727	0,9551	0,1974	
vehicle_48h	0,0107	0,025	0,0095	0,0103	0,0992	0,5301	0,0384	0,8124	0,0414

Figure 3 F	Neutrophils	24 h		
p-value table	sham	PCI vehicle	PCI T-Lipo	PCI AS
PCI vehicle	0,36			
PCI T-Lipo	0,36	0,77		
PCI AS	0,77	0,53	0,36	
PCI T-LipoAS	0,82	0,77	0,77	0,77

Figure 3 F	Neutrophils	48 h		
p-value table	**sham**	**PCI vehicle**	**PCI T-Lipo**	**PCI AS**
**PCI vehicle**	0,37			
**PCI T-Lipo**	0,17	0,24		
**PCI AS**	48	0,33	0,31	
**PCI T-LipoAS**	0,31	0,33	0,33	0,48

Figure 3 G	Monocytes	24 h		
p-value table	**sham**	**PCI vehicle**	**PCI T-Lipo**	**PCI AS**
**PCI vehicle**	0,925			
**PCI T-Lipo**	0,059	0,610		
**PCI AS**	0,925	0,950	0,881	
**PCI T-LipoAS**	0,881	0,950	0,940	1,000

Figure 3 G	Monocytes	48 h		
p-value table	**sham**	**PCI vehicle**	**PCI T-Lipo**	**PCI AS**
**PCI vehicle**	0,77			
**PCI T-Lipo**	0,147	0,025		
**PCI AS**	0,788	0,147	0,147	
**PCI T-LipoAS**	0,788	0,788	0,147	0,788

Figure 3 H	PMN positive cells								
p-value table	**PCI_AS_24h**	**PCI_AS_48h**	**sham_24h**	**sham_48h**	**T-Lipo_24h**	**T-Lipo_48h**	**T-LipoAS_24h**	**T-LipoAS_48h**	**vehicle_24h**
**PCI_AS_48h**	0,0424								
**sham_24h**	1	0,016							
**sham_48h**	0,2721	0,2249	0,2249						
**T-Lipo_24h**	0,0336	0,1088	0,0176	0,0722					
**T-Lipo_48h**	0,0424	0,0714	0,0336	0,0536	0,9144				
**T-LipoAS_24h**	0,0189	0,2278	0,0094	0,0542	0,6797	0,9144			
**T-LipoAS_48h**	0,0336	1	0,009	0,2063	0,1785	0,1826	0,2249		
**sham**	0,2249	0,0039	0,2249	0,0747	0,009	0,0176	0,0014	0,0014	

Figure 4

Figure 4C	AUC indocyanine green clearance		
p-value table	**PCI LipoAS**	**PCI Vehicle**	**sham LipoAS**
**PCI Vehicle**	1,20E-06		
**sham LipoAS**	1,40E-05	2,40E-05	
**sham Vehicle**	0,11	1,40E-05	9,90E-05

Figure 5

Figure 5 B	Survival		
p-value table	**PCI Vehicle**	**PCI AS**	**sham Vehicle**
**PCI AS**	0,28622		
**sham Vehicle**	0,00011	0,00072	
**sham AS**	0,00072	0,00411	1

Figure 5 C	Survival			
p-value table	**PCI Vehicle**	**PCI T- Lipo**	**PCI T-LipoAS**	**sham T-LipoAS**
**PCI T-Lipo**	0,9135			
**PCI T-LipoAS**	0,0053	0,0053		
**sham T-LipoAS**	0,0028	0,003	0,0561	
**sham T-Lipo**	0,0103	0,0144	0,1479	1

Appendix Figure S3

Appendix Figure S3	Interferon 1 beta (IFN-1β)			
p-value table	**sham**	**PCI Vehicle**	**PCI T-Lipo**	**PCI AS**
**PCI Vehicle**	1,60E-03			
**PCI T-Lipo**	2,89E-02	0,2542		
**PCI AS**	2,89E-02	2,89E-02	0,847	
**PCI T-LipoAS**	7,50E-03	0,0674	0,8672	0,9049

Appendix Figure S3	Interferon 1 beta (IFN-1β)			
p-value table	**sham**	**PCI Vehicle**	**PCI T-Lipo**	**PCI AS**
**PCI Vehicle**	1,70E-02			
**PCI T-Lipo**	1,99E-01	0,335		
**PCI AS**	3,60E-01	0,039	0,379	
**PCI T-LipoAS**	3,60E-01	0,039	0,335	0,929

Appendix Figure S3	Interleukin 1 alpha (IL-1α)			
p-value table	**sham**	**PCI Vehicle**	**PCI T-Lipo**	**PCI AS**
**PCI Vehicle**	2,00E-02			
**PCI T-Lipo**	1,81E-01	0,045		
**PCI AS**	4,58E-01	0,055	0,807	
**PCI T-LipoAS**	1,00E-02	4,58E-01	0,04	0,036

Appendix Figure S5

Appendix Figure S5	Survival			
p-value table	**PCI Vehicle**	**PCI Lipo**	**PCI LipoAS**	**sham Lipo**
**PCI Lipo**	0,732			
**PCI LipoAS**	0,1764	0,2361		
**sham Lipo**	0,0014	0,0106	0,0014	
**sham LipoAS**	0,0888	0,1764	0,0888	1
