# Supplementary material for: Targeted delivery of a phosphoinositide 3‐kinase γ inhibitor to restore organ function in sepsis
Source: EMBO Mol Med. 2021 Sep 2;13(10):e14436. doi: 10.15252/emmm.202114436 (PMC8495460; doi:10.15252/emmm.202114436)
Supplement: Supplementary file 2 [file EMMM-13-e14436-s001.pdf]

EMBO Molecular Medicine

Targeted delivery of a phosphoinositide 3-kinase inhibitor to restore organ function in sepsis

Adrian Press, Petra Babic, Bianca Hoffmann, Tina Mueller, Wanling Foo, Walter Hauswald, Jovana Benecke, Martina Beretta, Zoltán Cseresnyés, Stephanie Hoeppner, Ivo Nischang, Sina Coldewey, Markus Graeler, Reinhard Bauer, Falk Gonnert, Nikolaus Gassler, Reinhard Wetzker, Thilo Figge, Ulrich Schubert, and Michael Bauer
DOI: 10.15252/emmm.202114436

Corresponding author: Michael Bauer (Michael.Bauer@med.uni-jena.de)

Review Timeline:

Transfer from Review Commons:	14th Apr 21
Editorial Decision:	16th Apr 21
Revision Received:	23rd Jun 21
Editorial Decision:	13th Jul 21
Revision Received:	11th Aug 21
Accepted:	12th Aug 21

Editor: Lise Roth

Review COMMONS

Transaction Report:

This manuscript was transferred to EMBO Molecular Medicine following peer review at Review Commons.
(Note: With the exception of the correction of typographical or spelling errors that could be a source of ambiguity, letters and reports are not edited. Depending on transfer agreements, referee reports obtained elsewhere may or may not be included in this compilation. Referee reports are anonymous unless the Referee chooses to sign their reports.)

Review #1

1. How much time do you estimate the authors will need to complete the suggested revisions:

Estimated time to Complete Revisions (Required)

(Decision Recommendation)

Less than 1 month

2. Evidence, reproducibility and clarity:

Evidence, reproducibility and clarity (Required)

The work presented here is based on a previous study of the group, using phosphatidylinositol 3-kinase γ (PI3Kγ) knockout mice in early sepsis. These mice were protected against hepatic excretory dysfunction during sepsis, but the global PI3Kγ knockout led to an inhibition of neutrophil migration, which is an important function to guarantee an appropriate immune response. Here, the authors wanted to specifically target PI3K-g in hepatic parenchymal cells without altering its function in immune cells. To achieve this, the authors used dye-functionalized liposomes. The used DY-635 is a fluorescent polymethine dye, which as well a ligand for organic anion transporters. Considering this, it can selectively deliver cargo, i.e. therapeutics, to hepatic parenchymal cells. However, the authors started the study with the determination of PI3Kγ expression in human liver and the effect of PI3Kγ global as well as liver specific knockout and systemic use of the PI3Kγ inhibitor AS604240 on mouse survival following peritoneal cavity infection with human stool. These experiments showed no difference in mouse survival with the global PI3Kγ knockout compared to wild type mice and no alteration systemically using PI3Kγ inhibitor compared to the vehicle control. In contrast, the liver specific PI3Kγ knockout showed a better survival at the early sepsis phase compared to the wild type. Based on these results, the authors were encouraged to develop a hepatocyte-specific pharmacological selective inhibition of PI3Kγ function.
After characterization of the used liposomes, Press et al. analyzed the effect of the liposomes compared to the systemically applied PI3Kγ inhibitor. With this setting, the authors found, that the liposomes prevented PI3Kγ inhibition in immune cells, thus maintaining a functional immune response, whereas the nanoformulated PI3Kγ inhibition restored excretory liver function in vivo. Finally, this hepatocyte specific PI3Kγ inhibition was associated with improved mouse survival.

However, some concerns should be addressed:

Graphical abstract

For the reader it is difficult to understand why in the upper part the figure the upper arrow is red, directed to the right and the lower is blue, directed to the left and in the lower part of the figure its vice versa. And why the upper read arrow is PI3Kγ activation and the lower blue arrow is PI3Kγ activation. Possibly, "inhibition" would be better not illustrated by an arrow.

In the manuscript, the authors mentioned that besides in the liver the nanoformulated PI3Kγ inhibitor also is accumulating in the kidney. What is the consequence? Please add at least a part in the Discussion section.

In the last paragraph of the Introduction, Press et al. used the word "avoiding" which seems here not to be correct.

Why is PCI used and not the gold standard CLP? Please comment!

Figure 1B: Why is H3 in lanes 1 and 2 one band and in lanes 3 and 4 a double band? Please explain!

Is uptake of apoptotic/necrotic hepatic parenchymal cells by macrophages a problem? Please explain!

3. Significance:

Significance (Required)

Considering the need of therapies for the treatment of sepsis, the work of Press et al. significantly adds to the development of new therapeutic settings, which might be translated to the sepsis patient

Referees cross-commenting

The comments of reviewers 2 und 3 are valid and should be considered appropriately.

Review #2

1. How much time do you estimate the authors will need to complete the suggested revisions:

Estimated time to Complete Revisions (Required)

(Decision Recommendation)

Less than 1 month

2. Evidence, reproducibility and clarity:

Evidence, reproducibility and clarity (Required)

The authors have undertaken an elegant exploration of whether small molecular drugs selectively delivered to the hepatic parenchyma is beneficial in sepsis, following on from their previous extensive work in the area. The biologic rationale and experimental execution are first rate. The detrimental effects of complete PI3Kgamma inhibition cause impaired neutrophil migration and delayed resolution of infection. The key conclusions are convincing.

Major comments:

1. The experiments adequately replicated and statistical analysis adequate- Some of the figure legends would benefit from adding numbers of animals/group and summary stats used [e.g. figure 4].

Minor comments:

The neutrophil/monocyte strategy for flow cytometry identification requires clarification and uses an unconventional approach- was Ly6G used as well?

3. Significance:

Significance (Required)

The significance of this work is that they have successfully dissociated the benefits of T-LipoAS whilst maintaining an intact immune response at the peritoneal site of infection. The successful pharmacokinetic characterization of DY-635 conjugated liposomal carrier which increases the bioavailability of the PI3Kγ inhibitor AS605240 compartmentalized to hepatocytes, significantly improved liver function and survival - this is a genuine breakthrough in this area. As a targeted thereapy, its represents a landmark preclinical study in the field of sepsis.

Review #3

1. How much time do you estimate the authors will need to complete the suggested revisions:

Estimated time to Complete Revisions (Required)

(Decision Recommendation)

Between 1 and 3 months

2. Evidence, reproducibility and clarity:

Evidence, reproducibility and clarity (Required)

The manuscript by Press et al. describes generation of a novel liver-specific formulation of the PI3K-gamma inhibitor in lipid nanocarrier. The authors have previously shown that activation of PI3K-gamma in hepatocytes contributes to sepsis-induced liver excretory dysfunction, while PI3K-gamma in immune cells is necessary for protective antibacterial response. In this current study, they extend their observations by analysis of human and liver samples showing parenchymal expression of PI3K. Also, they confirm that liver-specific knock-out of PI3K-gamma tends to improve survival in murine sepsis model while systemic delivery of PI3K-gamma inhibitor does not influence the outcome of sepsis. Then, effort was made to generate functionalized lipid nanocarriers that would specifically deliver the inhibitor into the liver parenchymal cells. This goal was achieved by synthesis of DPPE liposomes with a fluorescent dye DY-635 which is specifically up taken by hepatocytes. The newly generated liposomes were characterized in detail and with use of sophisticated imaging tools and its inhibitory effect on PI3K-gamma were confirmed. Importantly, hepatocyte-specific delivery of the new nanocarrier was confirmed. Treatment of septic mice in a clinically relevant model with the T-LipoAS decreased systemic cytokines concentrations while, in contrast to inhibitor alone, it did not impair the bacterial clearance from peritoneal cavity. By the means of intravital microscopy and multispectral optoacoustic tomography showed that treatment of septic mice with T-LipoAS improved structural and functional sepsis-induced hepatocyte dysfunction. In a well-designed pre-clinical treatment study, the T-LipoAS treatment was related with improved survival of septic mice. These findings provide a novel approach to organ-specific treatment in sepsis-induced liver dysfunction.

Major comments:

- It would be important to know pharmokinetics of the the T-LipoAS. What is the T1/2 of the inhibitor? The information whether the inhibitor binding to PI3K-gamma is irreversible is lacking. These data should be provided to assess the treatment regimen for pre-clinical trial.
- The authors conclusions on the immunomodulatory effects of T-LipoAS (p.16) seem unjustified. In order to claim that the reduction of blood cytokine concentration is secondary to local immunomodulation instead of systemic effects further experiments are needed, e.g. comparison of cytokine production by cells from peritoneal lavage with cells from distant sites known to contribute to a given cytokine production (e.g. spleen and lymph nodes for IFN-gamma). Actually, it has been conceptualized and shown (MID: 24992991, 22751621) that the major problem of maladaptive immune response in sepsis is its systemic rather than locally tailored character. The presented results

suggest the opposite: that similar bacterial counts and cellular infiltration in the treatment and vehicle group suggests that treatment did not impaired local immune response but rather the systemic inflammation. Whether and how it is regulated by the liver remains to be elucidated. One simple thing that could be done is evaluation of the response of Kupfer cells (by means of mRNA expression or protein for cytokines) in the treated group, since these liver-located cells are quite probable to benefit from hepatoprotective effects of T-LipoAS.
-As the Figure 5C shows, the mortality benefit in T-LipoAS treated mice appear from day 3 on after PCI it would be of great interest to compare the inflammatory response (at least systemic cytokine level and peritoneal cell count and phagocytic function) at this later timepoint. Even more importantly the influence of treatment of secretory liver functions should be analyzed. Early effects of treatment are likely to be responsible for these later effects, but it would be important in terms of mechanisms of mortality benefits

Minor:

- Information from the 1st {section sign} of discussion should be moved into introduction section since they are important to understand the background and justification of undertaking the whole study.
- Commentary to Fig 1D is not justified; lack of mortality difference does not mean that the knock-out has balanced effect.
- description fig 1D: number of mice should be given- this information should be provided in methods or results section too
- full names for OATs and OATPs should be given (p.10)
- were the effects of the nanocarrier drug on kidney determined? Since its accumulation was second highest in kidney and the effects of PI3Ky are cell-dependent it should be evaluated.
-figure 3, number of mice per group must be given, also the graph's descriptions are almost impossible to read
- Figure 3H: information on what sections are shown is missing
- Figure 5: number of mice per group is lacking
-number and sex of used animals in each experiment should be given.

3. Significance:

Significance (Required)

This study provides both conceptual and technical advance in the field of sepsis-induced liver dysfunction. The idea of functionalized and organ-specific nanocarriers has been previously developed by the authors (PMID: 25470305). In this study they combined this approach with a concept of PI3K-gamma-mediated liver dysfunction in sepsis, which they also developed previously. Here, they provide a proof-of concept type study which shows utility of the novel technology to target specific kinase involved into disease pathogenesis. Therefore, the study although not frontier, is an important step in development of new treatment strategy in sepsis. This study will be of special interest to basic and translational researchers working on new-drug delivery methods, theranostics, sepsis and infectious diseases and liver dysfunction.

This review is written from a position of specialist in intensive care and a researcher in the immunology of sepsis and infectious diseases. I declare lack of specialty knowledge in the field of liposome development and study.

Referees cross-commenting
I agree with all the comments by reviewers 1 and 2 and the authors should correct the manuscript accordingly to them.

Reviewer #1

- For the reader it is difficult to understand why in the upper part the figure the upper arrow is red, directed to the right and the lower is blue, directed to the left and in the lower part of the figure its vice versa. And why the upper read arrow is PI3Kγ activation and the lower blue arrow is PI3Kγ activation. Possibly, "inhibition" would be better not illustrated by an arrow.

Reply: We appreciate the advice of the reviewer and simplified the Figure according to the suggestions (see below).

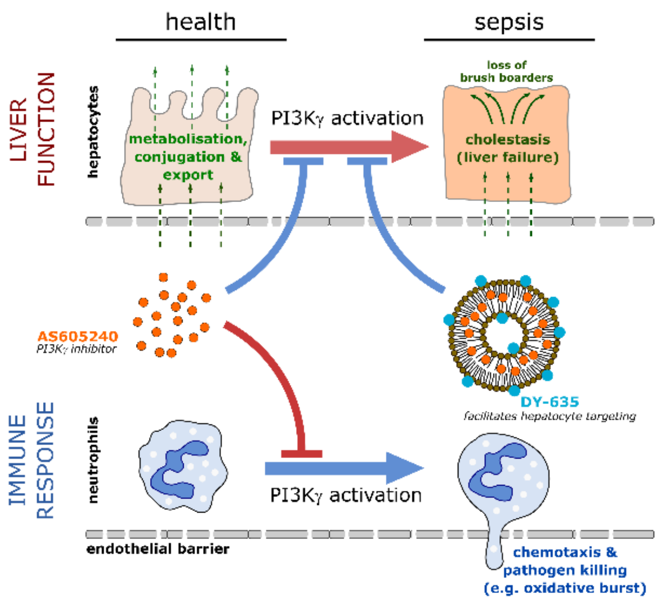

- In the manuscript, the authors mentioned that besides in the liver the nanoformulated PI3Kγ inhibitor also is accumulating in the kidney. What is the consequence? Please add at least a part in the Discussion section.

Reply: The possible consequences and potential causes of T-LipoAS accumulation in the kidney have been now considered in the Discussion part of the revised manuscript as requested.

- In the last paragraph of the Introduction, Press et al. used the word "avoiding" which seems here not to be correct.

Reply: We attenuated the statement in the revised manuscript to stay within the boundaries of our data.

- Why is PCI used and not the gold standard CLP? Please comment!

Reply: The reviewer specifically addresses the rationale for using „PCI (peritoneal contamination and infection)“ instead of CLP, as the specific sepsis model. We have characterized this model in detail in particular regarding molecular mechanisms of liver dysfunction and role of phosphatidylinositol-3-kinase signalling (Recknagel P, et al. PLoS Medicine. 2012;9(11):e1001338. Schaarschmidt B, et al. Theranostics. 2018 Jun 13;8(14):3766-3780.)

The model is compatible with the recommendations of a „Wiggers-Bernard Consensus Conference“ on “Pre-clinical Modeling in Sepsis: Exchanging Opinions and Forming Recommendations“ that led to a “Minimum Quality Threshold in Pre-Clinical Sepsis Studies (MQTiPSS) descriptor“. The participants of the consensus meeting identified and addressed several broad, critically important concepts in animal sepsis modeling. A total of 31 experts from 13 countries participated in the initiative (including five members of the Sepsis-3 definitions task force) and our model meets requirements regarding (1) study design, (2) humane endpoints, (3) infection types, (4) organ failure/dysfunction, (5) critical fluid resuscitation, and (6) antimicrobial therapy: Osuchowski MF et al. Minimum Quality Threshold in Pre-Clinical Sepsis Studies (MQTiPSS): An International Expert Consensus Initiative for Improvement of Animal Modeling in Sepsis. Shock. 2018 Oct;50(4):377-380. doi: 10.1097/SHK.0000000000001212.

- Figure 1B: Why is H3 in lanes 1 and 2 one band and in lanes 3 and 4 a double band? Please explain!

Reply: The H3 double band had been previously identified as two different H3 isoforms - H3a (upper band) and H3b (lower band); e.g. Hsu 2012 et al. (Nuc Acid Res. 40(15):7242-56, PMID: 22600736). The antibody used was targeted to a homologous domain. The double band suggests a different expression of H3 isoforms in different cell types. However these findings, though interesting, had not been the focus of this study and further evidence has to be generated to draw a conclusions and exclude methodological artifacts.

Is uptake of apoptotic/necrotic hepatic parenchymal cells by macrophages a problem? Please explain!

Reply: The release of the cellular content of apoptotic or necrotic hepatocytes may lead to the uptake of cell debris in macrophages and other non-parenchymal cells. As a consequence, immune cells might exhibit inflammatory responses. In the setting of our study, T-LipoAS has been found to provoke protective effects on hepatic parenchymal cells hence preventing significant stimulation of immune cells via apoptotic/necrotic hepatic parenchymal cells. This important point by the referee has now been added in the discussion.

Reviewer #2

The authors have undertaken an elegant exploration of whether small molecular drugs selectively delivered to the hepatic parenchyma is beneficial in sepsis, following on from their previous extensive work in the area. The biologic rationale and experimental execution are first rate. The detrimental effects of complete PI3Kgamma inhibition cause impaired neutrophil migration and delayed resolution of infection. The key conclusions are convincing.

We appreciate this positive assessment of our work by the reviewer.

Major comments:

- The experiments adequately replicated and statistical analysis adequate- Some of the figure legends would benefit from adding numbers of animals/group and summary stats used [e.g. figure 4].

Reply: Additional statistical information and group sizes are now provided in the new Supplementary Information 2.

Minor comments:

- The neutrophil/monocyte strategy for flow cytometry identification requires clarification and uses an unconventional approach- was Ly6G used as well?

Reply: The staining/gating of the monocytes and neutrophils cells was performed as previously described by Watson et al. (J Immunol. 2016, 194(6): 2796-2809, PMID: 25681345). The cell populations (neutrophils/monocytes) gated by this approach reflect a global infiltration pattern of all CD11b/CD45 positive cells. The gating strategy is now mentioned in the method section and provided in the Supplementary Information 1 (as new Figure S6).

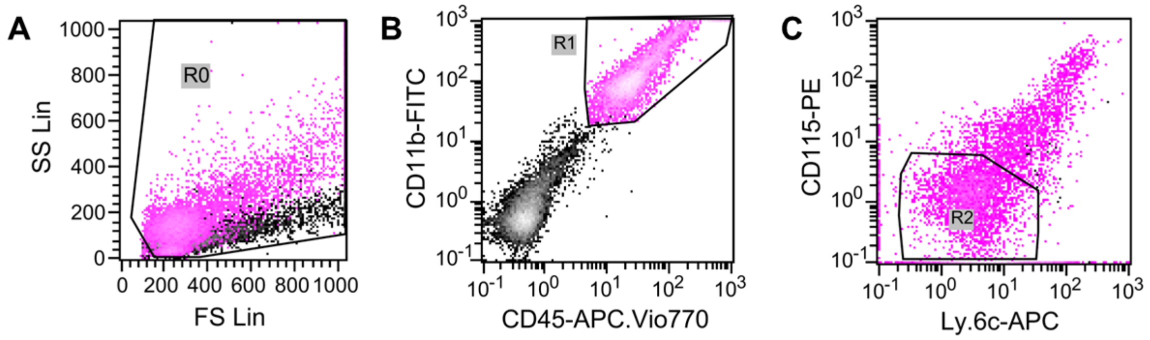

Figure S6: Gating of Lavage cells for analysis. Gating was performed as previously described by Watson et al. (J Immunol. 2016, 194(6): 2796-2809, PMID: 25681345). Peritoneal lavage as well as subsequent processing and staining was carried out as described in the method section. The figure depicts gating-strategy. A stained (pink) and unstained (black/grey) sample is overlaid in the XY-Plots: (**A**) Forward scatter vs. side scatter plot with gate R0 to separate cell events from debris. (**B**) Fluorescence labeled double positive CD11b+ CD45+ cells are then gated (R1) from R0. (**C**) CD115- and Ly-6c negative fraction of R1 is depicted and R2 gate is used to count cells neutrophils. CD115+, Ly6c+ cells (#R1-#R2, with # stands for the number of cells in each gate) are classified as monocytes. Data are normalized to the volume used for measurement obtaining the concentration of cells per volume lavage.

Reviewer #3

The manuscript by Press et al. describes generation of a novel liver-specific formulation of the PI3K-gamma inhibitor in lipid nanocarrier. The authors have

previously shown that activation of PI3K-gamma in hepatocytes contributes to sepsis-induced liver excretory dysfunction, while PI3K-gamma in immune cells is necessary for protective antibacterial response. In this current study, they extend their observations by analysis of human and liver samples showing parenchymal expression of PI3K. Also, they confirm that liver-specific knock-out of PI3K-gamma tends to improve survival in murine sepsis model while systemic delivery of PI3K-gamma inhibitor does not influence the outcome of sepsis. Then, effort was made to generate functionalized lipid nanocarriers that would specifically deliver the inhibitor into the liver parenchymal cells. This goal was achieved by synthesis of DPPE liposomes with a fluorescent dye DY-635 which is specifically up taken by hepatocytes. The newly generated liposomes were characterized in detail and with use of sophisticated imaging tools and its inhibitory effect on PI3K-gamma were confirmed. Importantly, hepatocyte-specific delivery of the new nanocarrier was confirmed. Treatment of septic mice in a clinically relevant model with the T-LipoAS decreased systemic cytokines concentrations while, in contrast to inhibitor alone, it did not impair the bacterial clearance from peritoneal cavity. By the means of intravital microscopy and multispectral optoacoustic tomography showed that treatment of septic mice with T-LipoAS improved structural and functional sepsis-induced hepatocyte dysfunction. In a well-designed pre-clinical treatment study, the T-LipoAS treatment was related with improved survival of septic mice. These findings provide a novel approach to organ-specific treatment in sepsis-induced liver dysfunction.

We appreciate this positive assessment of our work by the reviewer.

Major comments:

- It would be important to know pharmokinetics of the the T-LipoAS. What is the T1/2 of the inhibitor? The information whether the inhibitor binding to PI3K-gamma is irreversible is lacking. These data should be provided to assess the treatment regimen for pre-clinical trial.

Reply: Respective animal protocols had been submitted to the local authorities. Currently we await for the ethical and governmental approval and will contribute PDR-data for T-LipoAS. This information will asap be added in the revision process.

- The authors conclusions on the immunomodulatory effects of T-LipoAS (p.16) seem unjustified. In order to claim that the reduction of blood cytokine concentration is secondary to local immunomodulation instead of systemic effects further experiments are needed, e.g. comparison of cytokine production by cells from peritoneal lavage with cells from distant sites known to contribute to a given cytokine production (e.g. spleen and lymph nodes for IFN-gamma). Actually, it has been conceptualized and shown (MID: 24992991, 22751621) that the major problem of maladaptive immune response in sepsis is its systemic rather than locally tailored character. The presented results suggest the opposite: that similar bacterial counts and cellular infiltration in the treatment and vehicle group suggests that treatment did not impaired local immune response but rather the systemic inflammation. Whether and how it is regulated by the liver remains to be elucidated. One simple thing that could be

done is evaluation of the response of Kupfer cells (by means of mRNA expression or protein for cytokines) in the treated group, since these liver-located cells are quite probable to benefit from hepatoprotective effects of T-LipoAS.

Reply: We apologize for the mistaken depiction of the immunomodulatory effects of T-LipoAS on p. 16. Summarizing the experimental results presented in Fig. 3 our data clearly reveal that T-LipoAS does not substantially reduce blood cytokine concentration in comparison to vehicle- and T-Lipo-treated PCI mice; thus, similar results could be conceptualized by effects of PI3Kγ on the gradient between plasma and peritoneum. However, a relative decrease of blood cytokine level in the presence of T-LipoAS becomes only apparent in relation to mice treated with free AS. Consequently, on the basis of the data we do not see evidence for a claim that the reduction of blood cytokine concentration is secondary to local immunomodulation. Free AS, as reported in recent studies, expresses ability to induce strong increase of blood cytokines just by systemic inhibition of PI3Kgamma. In our setting free AS is provoking strong increase of cytokines in comparison to either vehicle or T-LipoAS treated PCI mice. In addition, the free inhibitor exhibits pronounced ability to inhibit the migration of blood phagocytes and consequent clearance of bacteria. Both systemic immune effects of the free inhibitor are masked by nanoformulation in T-LipoAS corroborating the central goal of our study. Following this adapted interpretation of the results shown in Fig. 3 we revised the text accordingly.

- As the Figure 5C shows, the mortality benefit in T-LipoAS treated mice appear from day 3 on after PCI it would be of great interest to compare the inflammatory response (at least systemic cytokine level and peritoneal cell count and phagocytic function) at this later timepoint. Even more importantly the influence of treatment of secretory liver functions should be analyzed. Early effects of treatment are likely to be responsible for these later effects, but it would be important in terms of mechanisms of mortality benefits

Reply: We agree with the reviewer that later effects of T-LipoAS would considerably strengthen insights in the mechanisms of mortality benefits, but we may reveal the enormous effort inevitable for prolonged experiments due to the high mortality of the control (vehicle) animals. Experiments deepening the mechanistic insights in the pathological functions of PI3Kγ in liver and kidney inflammatory diseases are currently underway in our laboratory and topic of a follow up study.

Minor:

- Information from the 1st {section sign} of discussion should be moved into introduction section since they are important to understand the background and justification of undertaking the whole study.

Reply: We appreciate the feedback on our manuscript and had moved the contents of the first paragraph of the discussion to the introduction.

- Commentary to Fig 1D is not justified; lack of mortality difference does not mean that the knock-out has balanced effect.

Reply: We agree with the reviewer, that this interpretation cannot be concluded solely from the experimental data provided. Therefore, we removed the statement.

- description fig 1D: number of mice should be given- this information should be provided in methods or results section too

Reply: A summarized statement on the number of replicates is given now in the figure legend for the survival analysis. Additional statistical information and detailed group sizes (incl. gender-distributions) are now provided in the new Supplementary Information 2.

- full names for OATs and OATPs should be given (p.10)

Reply: The abbreviations are now moved directly behind the fully spelled terms.

- were the effects of the nanocarrier drug on kidney determined? Since its accumulation was second highest in kidney and the effects of PI3Kγ are cell-dependent it should be evaluated.

Reply: The possible consequences of T-LipoAS accumulation in the kidney have been now considered in the Discussion part of the revised manuscript.

- figure 3, number of mice per group must be given, also the graph's descriptions are almost impossible to read
  - Figure 3H: information on what sections are shown is missing
  - Figure 5: number of mice per group is lacking
  -number and sex of used animals in each experiment should be given.

Reply: We revised the Figure 3 and its legend to improve clarity. And specified the tissue section (liver) which had been analyzed in Figure 3H.

For Figure 5 a summarized statement on the number of replicates are given now in the figure legend for the survival analysis. Additional statistical information and detailed group sizes (incl. gender-distributions) are now provided in the new Supplementary Information 2.

1st Editorial Decision 16th Apr 2021

16th Apr 2021

Dear Dr. Bauer,

Thank you for the submission of your manuscript to our editorial offices. I have now had the opportunity to read it, together with the referees' reports and your rebuttal letter, and to discuss them with the other members of our editorial team.

We agree that the study fits the scope of the journal, and we appreciate that you addressed most of the points raised by the reviewers. We thus encourage you to submit a revised version of your manuscript to our office, including the modifications and revisions described in your point-by-point letter. Acceptance of the manuscript will entail a second round of review. EMBO Molecular Medicine encourages a single round of revision only and therefore, acceptance or rejection of the manuscript will depend on the completeness of your responses included in the next, final version of the manuscript. For this reason, and to save you from any frustrations in the end, I would strongly advise against returning an incomplete revision.

***

When submitting your revised manuscript, please carefully review the instructions that follow below. Failure to include requested items will delay the evaluation of your revision:

1) A .docx formatted version of the manuscript text (including legends for main figures, EV figures and tables). Please make sure that the changes are highlighted to be clearly visible.

2) Individual production quality figure files as .eps, .tif, .jpg (one file per figure).

3) A .docx formatted letter INCLUDING the reviewers' reports and your detailed point-by-point responses to their comments. As part of the EMBO Press transparent editorial process, the point-by-point response is part of the Review Process File (RPF), which will be published alongside your paper.

4) A complete author checklist, which you can download from our author guidelines (https://www.embopress.org/page/journal/17574684/authorguide#submissionofrevisions). Please insert information in the checklist that is also reflected in the manuscript. The completed author checklist will also be part of the RPF.

5) Before submitting your revision, primary datasets produced in this study need to be deposited in an appropriate public database (see
https://www.embopress.org/page/journal/17574684/authorguide#dataavailability).
Please remember to provide a reviewer password if the datasets are not yet public.
The accession numbers and database should be listed in a formal "Data Availability " section (placed after Materials & Method). Please note that the Data Availability Section is restricted to new primary data that are part of this study.

6) We would also encourage you to include the source data for figure panels that show essential data. Numerical data should be provided as individual .xls or .csv files (including a tab describing the

data). For blots or microscopy, uncropped images should be submitted (using a zip archive if multiple images need to be supplied for one panel). Additional information on source data and instruction on how to label the files are available at
.

7) Our journal encourages inclusion of *data citations in the reference list* to directly cite datasets that were re-used and obtained from public databases. Data citations in the article text are distinct from normal bibliographical citations and should directly link to the database records from which the data can be accessed. In the main text, data citations are formatted as follows: "Data ref: Smith et al, 2001" or "Data ref: NCBI Sequence Read Archive PRJNA342805, 2017". In the Reference list, data citations must be labeled with "[DATASET]". A data reference must provide the database name, accession number/identifiers and a resolvable link to the landing page from which the data can be accessed at the end of the reference. Further instructions are available at .

8) We replaced Supplementary Information with Expanded View (EV) Figures and Tables that are collapsible/expandable online. A maximum of 5 EV Figures can be typeset. EV Figures should be cited as 'Figure EV1, Figure EV2" etc... in the text and their respective legends should be included in the main text after the legends of regular figures.

- For the figures that you do NOT wish to display as Expanded View figures, they should be bundled together with their legends in a single PDF file called *Appendix*, which should start with a short Table of Content. Appendix figures should be referred to in the main text as: "Appendix Figure S1, Appendix Figure S2" etc.

- Additional Tables/Datasets should be labeled and referred to as Table EV1, Dataset EV1, etc. Legends have to be provided in a separate tab in case of .xls files. Alternatively, the legend can be supplied as a separate text file (README) and zipped together with the Table/Dataset file.
See detailed instructions here:
.

9) The paper explained: EMBO Molecular Medicine articles are accompanied by a summary of the articles to emphasize the major findings in the paper and their medical implications for the non-specialist reader. Please provide a draft summary of your article highlighting
- the medical issue you are addressing,
- the results obtained and
- their clinical impact.

This may be edited to ensure that readers understand the significance and context of the research. Please refer to any of our published articles for an example.

10) For more information: There is space at the end of each article to list relevant web links for further consultation by our readers. Could you identify some relevant ones and provide such information as well? Some examples are patient associations, relevant databases, OMIM/proteins/genes links, author's websites, etc...

11) Every published paper now includes a 'Synopsis' to further enhance discoverability. Synopses are displayed on the journal webpage and are freely accessible to all readers. They include a short stand first (maximum of 300 characters, including space) as well as 2-5 one-sentences bullet points that summarizes the paper. Please write the bullet points to summarize the key NEW findings. They should be designed to be complementary to the abstract - i.e. not repeat the same text. We

encourage inclusion of key acronyms and quantitative information (maximum of 30 words / bullet point). Please use the passive voice. Please attach these in a separate file or send them by email, we will incorporate them accordingly.

Please also suggest a striking image or visual abstract to illustrate your article as a png file 550 px-wide x 400-px high.

12) As part of the EMBO Publications transparent editorial process initiative (see our Editorial at http://embomolmed.embopress.org/content/2/9/329), EMBO Molecular Medicine will publish online a Review Process File (RPF) to accompany accepted manuscripts.
In the event of acceptance, this file will be published in conjunction with your paper and will include the anonymous referee reports, your point-by-point response and all pertinent correspondence relating to the manuscript. Let us know whether you agree with the publication of the RPF and as here, if you want to remove or not any figures from it prior to publication.
Please note that the Authors checklist will be published at the end of the RPF.

EMBO Molecular Medicine has a "scooping protection" policy, whereby similar findings that are published by others during review or revision are not a criterion for rejection. Should you decide to submit a revised version, I do ask that you get in touch after three months if you have not completed it, to update us on the status.

I look forward to receiving your revised manuscript.

Yours sincerely,

Lise Roth

Lise Roth, PhD
Editor
EMBO Molecular Medicine

To submit your manuscript, please follow this link:

Link Not Available

*Additional important information regarding Figures

Each figure should be given in a separate file and should have the following resolution:
Graphs 800-1,200 DPI
Photos 400-800 DPI
Colour (only CMYK) 300-400 DPI"

Figures are not edited by the production team. All lettering should be the same size and style; figure panels should be indicated by capital letters (A, B, C etc). Gridlines are not allowed except for log plots. Figures should be numbered in the order of their appearance in the text with Arabic numerals. Each Figure must have a separate legend and a caption is needed for each panel.

*Additional important information regarding figures and illustrations can be found at https://embomolmed.embopress.org/authorguide#figures

***

Rev_Com_number: RC-2021-00627
New_manu_number: EMM-2021-14436
Corr_author: Bauer
Title: Targeted delivery of a phosphoinositide 3-kinase γ inhibitor to restore organ function in sepsis through dye-functionalized lipid nanocarriers

Reviewer #1

- For the reader it is difficult to understand why in the upper part the figure the upper arrow is red, directed to the right and the lower is blue, directed to the left and in the lower part of the figure its vice versa. And why the upper read arrow is PI3Kγ activation and the lower blue arrow is PI3Kγ activation. Possibly, "inhibition" would be better not illustrated by an arrow.

Reply: We appreciate the advice of the reviewer and simplified the Figure according to the suggestions (see below).

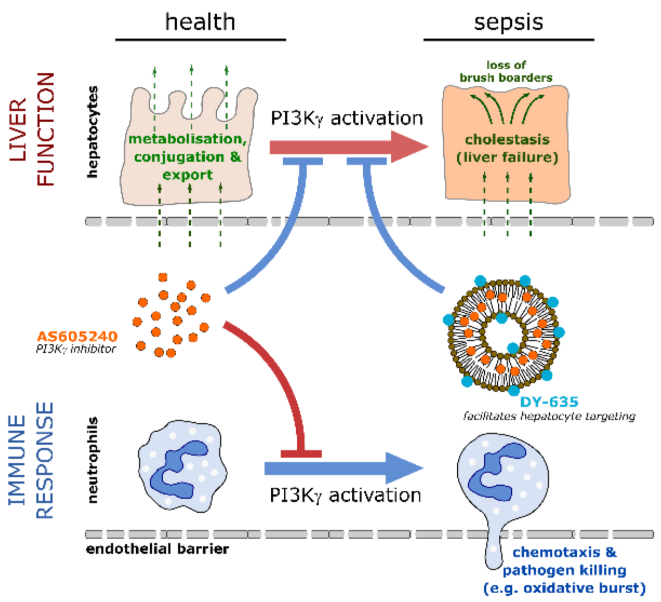

- In the manuscript, the authors mentioned that besides in the liver the nanoformulated PI3Kγ inhibitor also is accumulating in the kidney. What is the consequence? Please add at least a part in the Discussion section.

Reply: The possible consequences and potential causes of T-LipoAS accumulation in the kidney have been now considered in the Discussion part of the revised manuscript as requested.

- In the last paragraph of the Introduction, Press et al. used the word "avoiding" which seems here not to be correct.

Reply: We attenuated the statement in the revised manuscript to stay within the boundaries of our data.

- Why is PCI used and not the gold standard CLP? Please comment!

Reply: The reviewer specifically addresses the rationale for using „PCI (peritoneal contamination and infection)" instead of CLP, as the specific sepsis model. We have characterized this model in detail in particular regarding molecular mechanisms of liver dysfunction and role of phosphatidylinositol-3-kinase signalling (Recknagel P, et al. PLoS Medicine. 2012;9(11):e1001338. Schaarschmidt B, et al. Theranostics. 2018 Jun 13;8(14):3766-3780.)

The model is compatible with the recommendations of a „Wiggers-Bernard Consensus Conference“ on “Pre-clinical Modeling in Sepsis: Exchanging Opinions and Forming Recommendations“ that led to a “Minimum Quality Threshold in Pre-Clinical Sepsis Studies (MQTiPSS) descriptor“. The participants of the consensus meeting identified and addressed several broad, critically important concepts in animal sepsis modeling. A total of 31 experts from 13 countries participated in the initiative (including five members of the Sepsis-3 definitions task force) and our model meets requirements regarding (1) study design, (2) humane endpoints, (3) infection types, (4) organ failure/dysfunction, (5) critical fluid resuscitation, and (6) antimicrobial therapy: Osuchowski MF et al. Minimum Quality Threshold in Pre-Clinical Sepsis Studies (MQTiPSS): An International Expert Consensus Initiative for Improvement of Animal Modeling in Sepsis. Shock. 2018 Oct;50(4):377-380. doi: 10.1097/SHK.0000000000001212.

- Figure 1B: Why is H3 in lanes 1 and 2 one band and in lanes 3 and 4 a double band? Please explain!

Reply: The H3 double band had been previously identified as two different H3 isoforms - H3a (upper band) and H3b (lower band); e.g. Hsu 2012 et al. (Nuc Acid Res. 40(15):7242-56, PMID: 22600736). The antibody used was targeted to a homologous domain. The double band suggests a different expression of H3 isoforms in different cell types. However these findings, though interesting, had not been the focus of this study and further evidence has to be generated to draw a conclusions and exclude methodological artifacts.

Is uptake of apoptotic/necrotic hepatic parenchymal cells by macrophages a problem? Please explain!

Reply: The release of the cellular content of apoptotic or necrotic hepatocytes may lead to the uptake of cell debris in macrophages and other non-parenchymal cells. As a consequence, immune cells might exhibit inflammatory responses. In the setting of our study, T-LipoAS has been found to provoke protective effects on hepatic parenchymal cells hence preventing significant stimulation of immune cells via apoptotic/necrotic hepatic parenchymal cells. This important point by the referee has now been added in the discussion.

Reviewer #2

The authors have undertaken an elegant exploration of whether small molecular drugs selectively delivered to the hepatic parenchyma is beneficial in sepsis, following on from their previous extensive work in the area. The biologic rationale and experimental execution are first rate. The detrimental effects of complete PI3Kgamma inhibition cause impaired neutrophil migration and delayed resolution of infection. The key conclusions are convincing.

We appreciate this positive assessment of our work by the reviewer.

Major comments:

- The experiments adequately replicated and statistical analysis adequate- Some of the figure legends would benefit from adding numbers of animals/group and summary stats used [e.g. figure 4].

Reply: Additional statistical information and group sizes are now provided in the new Supplementary Information 2.

Minor comments:

- The neutrophil/monocyte strategy for flow cytometry identification requires clarification and uses an unconventional approach- was Ly6G used as well?

Reply: The staining/gating of the monocytes and neutrophils cells was performed as previously described by Watson et al. (J Immunol. 2016, 194(6): 2796-2809, PMID: 25681345). The cell populations (neutrophils/monocytes) gated by this approach reflect a global infiltration pattern of all CD11b/CD45 positive cells. The gating strategy is now mentioned in the method section and provided in the Supplementary Information 1 (as new Figure S6).

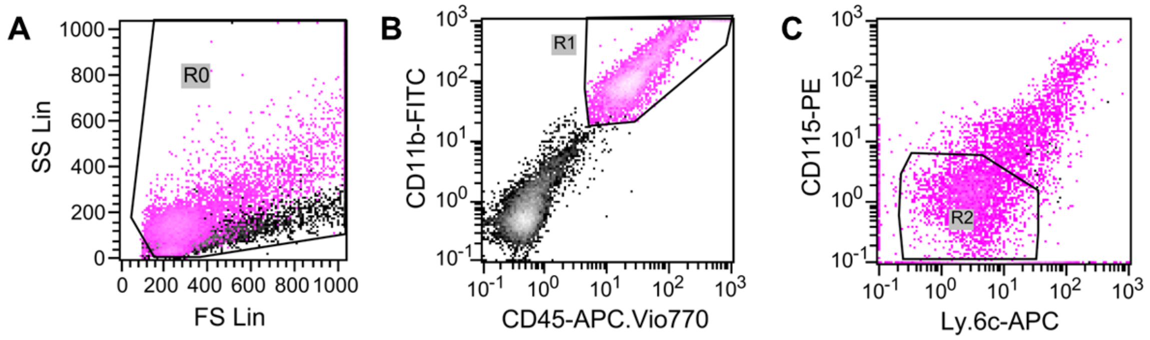

Figure S6: Gating of Lavage cells for analysis. Gating was performed as previously described by Watson et al. (J Immunol. 2016, 194(6): 2796-2809, PMID: 25681345). Peritoneal lavage as well as subsequent processing and staining was carried out as described in the method section. The figure depicts gating-strategy. A stained (pink) and unstained (black/grey) sample is overlaid in the XY-Plots: (**A**) Forward scatter vs. side scatter plot with gate R0 to separate cell events from debris. (**B**) Fluorescence labeled double positive CD11b+ CD45+ cells are then gated (R1) from R0. (**C**) CD115- and Ly-6c negative fraction of R1 is depicted and R2 gate is used to count cells neutrophils. CD115+, Ly6c+ cells (#R1-#R2, with # stands for the number of cells in each gate) are classified as monocytes. Data are normalized to the volume used for measurement obtaining the concentration of cells per volume lavage.

Reviewer #3

The manuscript by Press et al. describes generation of a novel liver-specific formulation of the PI3K-gamma inhibitor in lipid nanocarrier. The authors have previously shown that activation of PI3K-gamma in hepatocytes contributes to sepsis-induced liver excretory dysfunction, while PI3K-gamma in immune cells is necessary for protective antibacterial response. In this current study, they extend their observations by analysis of human and liver samples showing parenchymal expression of PI3K. Also, they confirm that liver-specific knock-out of PI3K-gamma

tends to improve survival in murine sepsis model while systemic delivery of PI3K-gamma inhibitor does not influence the outcome of sepsis. Then, effort was made to generate functionalized lipid nanocarriers that would specifically deliver the inhibitor into the liver parenchymal cells. This goal was achieved by synthesis of DPPE liposomes with a fluorescent dye DY-635 which is specifically up taken by hepatocytes. The newly generated liposomes were characterized in detail and with use of sophisticated imaging tools and its inhibitory effect on PI3K-gamma were confirmed. Importantly, hepatocyte-specific delivery of the new nanocarrier was confirmed. Treatment of septic mice in a clinically relevant model with the T-LipoAS decreased systemic cytokines concentrations while, in contrast to inhibitor alone, it did not impair the bacterial clearance from peritoneal cavity. By the means of intravital microscopy and multispectral optoacoustic tomography showed that treatment of septic mice with T-LipoAS improved structural and functional sepsis-induced hepatocyte dysfunction. In a well-designed pre-clinical treatment study, the T-LipoAS treatment was related with improved survival of septic mice. These findings provide a novel approach to organ-specific treatment in sepsis-induced liver dysfunction.

We appreciate this positive assessment of our work by the reviewer.

Major comments:

- It would be important to know pharmokinetics of the the T-LipoAS. What is the T1/2 of the inhibitor? The information whether the inhibitor binding to PI3K-gamma is irreversible is lacking. These data should be provided to assess the treatment regimen for pre-clinical trial.

Reply: The t1/2 of T-LipoAS had been estimated from large ear veins in mice. The fluorescence of DY-635 from T-LipoAS had been analyzed by intravital microscopy over a period of circa 45 min upon intravenous injection (tail vein) of the nanocarriers. The t1/2 = 55.9 min +/- 7.9 min had been calculated from the linear phase of the curve. The data are added as Figure 2D of the revised manuscript.

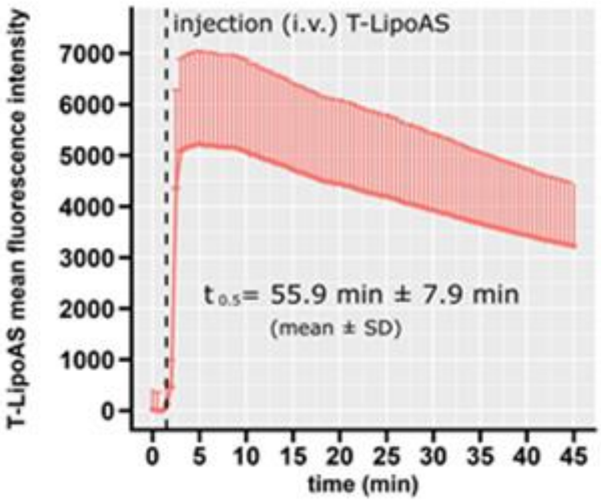

Figure 2D: T-LipoAS plasma disappearance rate in murine ear veins and estimated half-life obtained by intravital microscopy. (n=4)

- The authors conclusions on the immunomodulatory effects of T-LipoAS (p.16) seem unjustified. In order to claim that the reduction of blood cytokine

concentration is secondary to local immunomodulation instead of systemic effects further experiments are needed, e.g. comparison of cytokine production by cells from peritoneal lavage with cells from distant sites known to contribute to a given cytokine production (e.g. spleen and lymph nodes for IFN-gamma). Actually, it has been conceptualized and shown (MID: 24992991, 22751621) that the major problem of maladaptive immune response in sepsis is its systemic rather than locally tailored character. The presented results suggest the opposite: that similar bacterial counts and cellular infiltration in the treatment and vehicle group suggests that treatment did not impaired local immune response but rather the systemic inflammation. Whether and how it is regulated by the liver remains to be elucidated. One simple thing that could be done is evaluation of the response of Kupfer cells (by means of mRNA expression or protein for cytokines) in the treated group, since these liver-located cells are quite probable to benefit from hepatoprotective effects of T-LipoAS.

Reply: We apologize for the mistaken depiction of the immunomodulatory effects of T-LipoAS on p. 16. Summarizing the experimental results presented in Fig. 3 our data clearly reveal that T-LipoAS does not substantially reduce blood cytokine concentration in comparison to vehicle- and T-Lipo-treated PCI mice; thus, similar results could be conceptualized by effects of PI3Kγ on the gradient between plasma and peritoneum. However, a relative decrease of blood cytokine level in the presence of T-LipoAS becomes only apparent in relation to mice treated with free AS. Consequently, on the basis of the data we do not see evidence for a claim that the reduction of blood cytokine concentration is secondary to local immunomodulation. Free AS, as reported in recent studies, expresses ability to induce strong increase of blood cytokines just by systemic inhibition of PI3Kgamma. In our setting free AS is provoking strong increase of cytokines in comparison to either vehicle or T-LipoAS treated PCI mice. In addition, the free inhibitor exhibits pronounced ability to inhibit the migration of blood phagocytes and consequent clearance of bacteria. Both systemic immune effects of the free inhibitor are masked by nanoformulation in T-LipoAS corroborating the central goal of our study. Following this adapted interpretation of the results shown in Fig. 3 we revised the text accordingly.

- As the Figure 5C shows, the mortality benefit in T-LipoAS treated mice appear from day 3 on after PCI it would be of great interest to compare the inflammatory response (at least systemic cytokine level and peritoneal cell count and phagocytic function) at this later timepoint. Even more importantly the influence of treatment of secretory liver functions should be analyzed. Early effects of treatment are likely to be responsible for these later effects, but it would be important in terms of mechanisms of mortality benefits

Reply: We agree with the reviewer that later effects of T-LipoAS would considerably strengthen insights in the mechanisms of mortality benefits, but we may reveal the enormous effort inevitable for prolonged experiments due to the high mortality of the control (vehicle) animals. Experiments deepening the mechanistic insights in the pathological functions of PI3Kγ in liver and kidney inflammatory diseases are currently underway in our laboratory and topic of a follow up study.

Minor:

- Information from the 1st {section sign} of discussion should be moved into introduction section since they are important to understand the background and justification of undertaking the whole study.

Reply: We appreciate the feedback on our manuscript and had moved the contents of the first paragraph of the discussion to the introduction.

- Commentary to Fig 1D is not justified; lack of mortality difference does not mean that the knock-out has balanced effect.

Reply: We agree with the reviewer, that this interpretation cannot be concluded solely from the experimental data provided. Therefore, we removed the statement.

- description fig 1D: number of mice should be given- this information should be provided in methods or results section too

Reply: A summarized statement on the number of replicates is given now in the figure legend for the survival analysis. Additional statistical information and detailed group sizes (incl. gender-distributions) are now provided in the new Supplementary Information 2.

- full names for OATs and OATPs should be given (p.10)

Reply: The abbreviations are now moved directly behind the fully spelled terms.

- were the effects of the nanocarrier drug on kidney determined? Since its accumulation was second highest in kidney and the effects of PI3Ky are cell-dependent it should be evaluated.

Reply: The possible consequences of T-LipoAS accumulation in the kidney have been now considered in the Discussion part of the revised manuscript.

- figure 3, number of mice per group must be given, also the graph's descriptions are almost impossible to read
  - Figure 3H: information on what sections are shown is missing
  - Figure 5: number of mice per group is lacking
  -number and sex of used animals in each experiment should be given.

Reply: We revised the Figure 3 and its legend to improve clarity. And specified the tissue section (liver) which had been analyzed in Figure 3H.

For Figure 5 a summarized statement on the number of replicates are given now in the figure legend for the survival analysis. Additional statistical information and detailed group sizes (incl. gender-distributions) are now provided in the new Supplementary Information 2.

1st Revision - Editorial Decision 13th Jul 2021

13th Jul 2021

Dear Prof. Bauer,

Thank you for the submission of your revised manuscript to EMBO Molecular Medicine. We have now received the enclosed report from two of the referees who had reviewed your original manuscript. As you will see, they are now supportive of publication, and I am therefore pleased to inform you that we will be able to accept your manuscript once the following editorial points will be addressed:

1/ Main manuscript text:
- Please address the queries from our data editors in track changes mode in the main manuscript file labelled 'Related manuscript file'. Please use this file for any further modification.
- Please remove the highlighted text, and only keep in track changes the new modifications.
- Please remove the figures from the main manuscript file and compile the main figure legends at the end of the manuscript text.
- Please remove the one sentence summary and the graphical abstract from the main manuscript file.
- Material and methods:
o Animals: please provide detailed husbandry conditions (access to food and light cycle).
o Human samples: include a statement that informed consent was obtained from all subjects and that the experiments conformed to the principles set out in the WMA Declaration of Helsinki and the Department of Health and Human Services Belmont Report.
- Thank you for providing a Data Availability section. Datasets must be publicly available, therefore please remove: "The password and all data are available from the authors upon reasonable request."
- Please merge the funding information with the Acknowledgements.
- Please update the reference format to have them in alphabetical order, and with 10 authors listed before et al.
- Please replace "Contribution" by "Author contributions".

2/ Figures and Appendix:
- Please upload each figure separately.
- Please indicate in the main and appendix figures or in their legends the exact n and p= values, not a range, along with the statistical test used.
- There is a document with 2 suppl. tables and 6 suppl. figures: please upload as an appendix file in PDF format, with a table of content, and correct the nomenclature (Appendix Table S1 etc. and Appendix Figure S1 etc). Please also update the nomenclature in the main manuscript text.
- The document Suppl. Information 2 with additions to the figure legends should be merged with the legends in the main manuscript, or complementary information provided in the Appendix.

3/ Checklist:
Please carefully check your checklist for typos.
Section F: please provide information in F/18 and F/19. Datasets must be made publicly available, please remove "provided upon reasonable request".

4/ The paper explained: EMBO Molecular Medicine articles are accompanied by a summary of the articles to emphasize the major findings in the paper and their medical implications for the non-specialist reader. Please provide a draft summary of your article highlighting
- the medical issue you are addressing,
- the results obtained and
- their clinical impact.

This may be edited to ensure that readers understand the significance and context of the research. Please refer to any of our published articles for an example.

5/ For more information: There is space at the end of each article to list relevant web links for further consultation by our readers. Could you identify some relevant ones and provide such information as well? Some examples are patient associations, relevant databases, OMIM/proteins/genes links, author's websites, etc...

6/ Thank you for providing a synopsis image. Please remove it from the main manuscript file and upload it separately as a jpeg/png/tiff file 550 px wide x 300-600 px high.
Please also provide a synopsis text, which should include a short stand first (maximum of 300 characters, including space) as well as 2-5 one-sentence bullet points, that summarizes the paper. Please write the bullet points to summarize the key NEW findings. They should be designed to be complementary to the abstract - i.e. not repeat the same text.

7/ As part of the EMBO Publications transparent editorial process initiative (see our Editorial at http://embomolmed.embopress.org/content/2/9/329), EMBO Molecular Medicine will publish online a Review Process File (RPF) to accompany accepted manuscripts.
This file will be published in conjunction with your paper and will include the anonymous referee reports, your point-by-point response and all pertinent correspondence relating to the manuscript.
Let us know whether you agree with the publication of the RPF and as here, OR IF YOU WANT TO REMOVE OR NOT any figures from it prior to publication.
Please note that the Authors checklist will be published at the end of the RPF.

I look forward to receiving your revised manuscript.

Yours sincerely,

Lise Roth

Lise Roth, PhD
Editor
EMBO Molecular Medicine

You can submit your revised files by logging onto our online manuscript tracking system or simply follow this link:

Link Not Available

***** Reviewer's comments *****

Referee #1 (Remarks for Author):

Thanks for clarifying the gating strategy. Most readers would be surprised to not see Ly6G labelling, given how easy this is to add to the panel, but the authors have justified their approach ad while suboptimal this is unlikely to change the conclusions in any substantive way.

Referee #2 (Remarks for Author):

The concerns of all reviewers have been carefully addressed.

***
Rev_Com_number: RC-2021-00627
New_manu_number: EMM-2021-14436-V2
Corr_author: Bauer
Title: Targeted delivery of a phosphoinositide 3-kinase inhibitor to restore organ function in sepsis

The authors performed the requested editorial changes.

Dear Dr. Diederich,
Thank you for your patients and support in addressing the editorial queries. Please find the point-to-point response to your latest queries below:

All changes in the main manuscript had been made in "tracked-changes mode". One more reference was added to the Method section (Schuck & Rossmanith 2000), where I am not sure if the tracked changes worked correctly.
The Supplementary information is uploaded as pdf (without highlighted changes) as requested.

1. Please remove the 1-sentence-summary from manuscript's title page.
Reply: the one-sentence summary is now removed

2. We were unable to find information on the exact p values for any of the figures. Please either add them to the relevant figure legends, or compile them in an additional table that can be added to the appendix (as "Appendix Table S[number]").
Reply: the new **Appendix Table S3** summarizes the precise p-values for the statistical comparisons in the study.

3. Please add a table of contents to the appendix.
Reply: a table of contents had now been added on page 2 of the appendix

4. We noticed that there is now a list of "extended figure legends" that was added to the appendix. Please merge this information with the main figure legends. The associated tables should stay in the appendix and be relabeled "Appendix Table S[number]".
Reply: all information had now been combined with the main figure legends.

5. Please remove the sentence "All data are available from the authors upon reasonable request" in the Data Availability section; please refer to our our rules on "availability of published material and data"
https://www.embopress.org/page/journal/17574684/authorguide#availabilityofpublishedmaterial

Reply: all relevant data required for the evaluation of the study are presented in the manuscript and are now deposited on a server of the HKI. Therefore, the data availability statement had been updated accordingly:

"All data required for the evaluation of the study are presented in the manuscript and deposited on a server of the Hans-Knoell Insitute Jena (https://asbdata.hki-jena.de/PressEtAl2021_EMBOMolMed)."

6. Thank you for providing a list of additional materials for the For More Information section. Please add this to the manuscript text, after the Author Contributions.
Reply: the web resources had been added at the end of the manuscript in a new section "For More Information".

Please do not hesitate to get back to us in case of questions occur.

Kind regards,
Michael Bauer

12th Aug 2021

Dear Prof. Bauer,

Thank you for submitting the revised manuscript files. We are pleased to inform you that your manuscript is now accepted for publication in EMBO Molecular Medicine!

Before we can send it to our publisher, please address the following:

1/ Remove "reasonable" from "available upon reasonable request".
2/ Provide The Paper Explained section. I note that you have provided a lays summary, however The Paper Explained must follow a specific structure:

- the medical issue you are addressing,
- the results obtained and
- their clinical impact.

This may be edited to ensure that readers understand the significance and context of the research. Please refer to any of our published articles for an example.

3/ Accept all changes.

Please send us your modified manuscript (and TPE) via email.

We would like to remind you that as part of the EMBO Publications transparent editorial process initiative, EMBO Molecular Medicine will publish a Review Process File online to accompany accepted manuscripts. If you do NOT want the file to be published or would like to exclude figures, please immediately inform the editorial office via e-mail.

Congratulations on your interesting work!

With kind regards,

Lise Roth

Lise Roth, Ph.D
Scientific Editor
EMBO Molecular Medicine

Follow us on Twitter @EmboMolMed
Sign up for eTOCs at embopress.org/alertsfeeds

EMBO PRESS

YOU MUST COMPLETE ALL CELLS WITH A PINK BACKGROUND ↓

PLEASE NOTE THAT THIS CHECKLIST WILL BE PUBLISHED ALONGSIDE YOUR PAPER

Corresponding Author Name: Michael Bauer

Journal Submitted to: EMBO Molecular Medicine

Manuscript Number: EMM-2021-14436-V2

Reporting Checklist For Life Sciences Articles (Rev. June 2017)

This checklist is used to ensure good reporting standards and to improve the reproducibility of published results. These guidelines are consistent with the Principles and Guidelines for Reporting Preclinical Research issued by the NIH in 2014. Please follow the journal's authorship guidelines in preparing your manuscript.

A- Figures
1. Data
The data shown in figures should satisfy the following conditions:

- ➔ the data were obtained and processed according to the field's best practice and are presented to reflect the results of the experiments in an accurate and unbiased manner.
- ➔ figure panels include only data points, measurements or observations that can be compared to each other in a scientifically meaningful way.
- ➔ graphs include clearly labeled error bars for independent experiments and sample sizes. Unless justified, error bars should not be shown for technical replicates.
- ➔ if n< 5, the individual data points from each experiment should be plotted and any statistical test employed should be justified
- ➔ Source Data should be included to report the data underlying graphs. Please follow the guidelines set out in the author ship guidelines on Data Presentation.

2. Captions

Each figure caption should contain the following information, for each panel where they are relevant:

- ➔ a specification of the experimental system investigated (eg cell line, species name).
- ➔ the assay(s) and method(s) used to carry out the reported observations and measurements
- ➔ an explicit mention of the biological and chemical entity(ies) that are being measured.
- ➔ an explicit mention of the biological and chemical entity(ies) that are altered/varied/perturbed in a controlled manner.

- ➔ the exact sample size (n) for each experimental group/condition, given as a number, not a range;
- ➔ a description of the sample collection allowing the reader to understand whether the samples represent technical or biological replicates (including how many animals, litters, cultures, etc.).
- ➔ a statement of how many times the experiment shown was independently replicated in the laboratory.
- ➔ definitions of statistical methods and measures:
  - common tests, such as t-test (please specify whether paired vs. unpaired), simple χ2 tests, Wilcoxon and Mann-Whitney tests, can be unambiguously identified by name only, but more complex techniques should be described in the methods section;
  - are tests one-sided or two-sided?
  - are there adjustments for multiple comparisons?
  - exact statistical test results, e.g., P values = x but not P values < x;
  - definition of 'center values' as median or average;
  - definition of error bars as s.d. or s.e.m.

Any descriptions too long for the figure legend should be included in the methods section and/or with the source data.

In the pink boxes below, please ensure that the answers to the following questions are reported in the manuscript itself. Every question should be answered. If the question is not relevant to your research, please write NA (non applicable). We encourage you to include a specific subsection in the methods section for statistics, reagents, animal models and human subjects.

USEFUL LINKS FOR COMPLETING THIS FORM

http://www.antibodypedia.com
http://1degreebio.org
http://www.equator-network.org/reporting-guidelines/improving-bioscience-research-repor
http://grants.nih.gov/grants/olaw/olaw.htm
http://www.mrc.ac.uk/Ourresearch/Ethicsresearchguidance/Useofanimals/index.htm
http://ClinicalTrials.gov
http://www.consort-statement.org
http://www.consort-statement.org/checklists/view/32-consort/66-title
http://www.equator-network.org/reporting-guidelines/reporting-recommendations-for-tumo
http://datadryad.org
http://figshare.com
http://www.ncbi.nlm.nih.gov/gap
http://www.ebi.ac.uk/ega
http://biomodels.net/
http://biomodels.net/miriam/
http://jjj.biochem.sun.ac.za
http://oba.od.nih.gov/biosecurity/biosecurity_documents.html
http://www.selectagents.gov/

B- Statistics and general methods

	Please fill out these boxes ↓ (Do not worry if you cannot see all your text once you press return)
1.a. How was the sample size chosen to ensure adequate power to detect a pre-specified effect size?	For initial investigation, the number of samples where kept low in cases where ethical concerns, like animal welfare, were applicable. As initial data became available, a Power analysis was performed to estimate the additional number of animals needed
1.b. For animal studies, include a statement about sample size estimate even if no statistical methods were used.	Number of replicates are based on ethical considerations and previous experiments with the applied sepsis model. Optoacoustic Tomographic Study: After initial exploratory experiments with 2-5 animals per group, a Power analysis was performed to find significant results at the level p<0.05 with a Power of 0.85. This Power analysis was approved by the Thuringian State Office for Consumer Protection as part of the ethical approval of the animal trials.
2. Describe inclusion/exclusion criteria if samples or animals were excluded from the analysis. Were the criteria pre-established?	No animals were excluded.
3. Were any steps taken to minimize the effects of subjective bias when allocating animals/samples to treatment (e.g. randomization procedure)? If yes, please describe.	Animals were assigned towards groups to control the age and gender distribution in animals suffering sepsis.
For animal studies, include a statement about randomization even if no randomization was used.	Animals were assigned towards groups to control the age and gender distribution in animals suffering sepsis.
4.a. Were any steps taken to minimize the effects of subjective bias during group allocation or/and when assessing results (e.g. blinding of the investigator)? If yes please describe.	Animals were assigned towards groups to obtain a homogeneous age and gender distribution. The scientist infected the animals was blinded to the treatment group.
4.b. For animal studies, include a statement about blinding even if no blinding was done	Animals were assigned towards groups to obtain a homogeneous age and gender distribution. The scientist infected the animals was blinded to the treatment group.
5. For every figure, are statistical tests justified as appropriate?	the information on statistical testing and justifications are given in the Figure Legend and Supplementary Information 2
Do the data meet the assumptions of the tests (e.g., normal distribution)? Describe any methods used to assess it.	Due to the low sample size, normality and equal variance was not assumed.
Is there an estimate of variation within each group of data?	no

Is the variance similar between the groups that are being statistically compared?	no

C- Reagents

6. To show that antibodies were profiled for use in the system under study (assay and species), provide a citation, catalog number and/or clone number, supplementary information or reference to an antibody validation profile. e.g., Antibodypedia (see link list at top right), 1DegreeBio (see link list at top right).	those information are provided in the method section of the manuscript
7. Identify the source of cell lines and report if they were recently authenticated (e.g., by STR profiling) and tested for mycoplasma contamination.	HepaRG cells had been purchased from BIOPREDIC INTERNATIONAL. No additional STR profiling had been performed. Cell lines are regularly tested for mycoplasma contamination. No mycoplasma contamination was apparent in the period where experiments had been performed.

* for all hyperlinks, please see the table at the top right of the document

D- Animal Models

8. Report species, strain, gender, age of animals and genetic modification status where applicable. Please detail housing and husbandry conditions and the source of animals.	the details of the strains, their sources and husbandry conditions are stated in the method section of the manuscript
9. For experiments involving live vertebrates, include a statement of compliance with ethical regulations and identify the committee(s) approving the experiments.	a statement is provided in the method section of the manuscript
10. We recommend consulting the ARRIVE guidelines (see link list at top right) (PLoS Biol. 8(6), e1000412, 2010) to ensure that other relevant aspects of animal studies are adequately reported. See author guidelines, under 'Reporting Guidelines'. See also: NIH (see link list at top right) and MRC (see link list at top right) recommendations. Please confirm compliance.	The Author Checklist for the arrive guidelines had been followed.

E- Human Subjects

11. Identify the committee(s) approving the study protocol.	Jena University Hospital
12. Include a statement confirming that informed consent was obtained from all subjects and that the experiments conformed to the principles set out in the WMA Declaration of Helsinki and the Department of Health and Human Services Belmont Report.	the use of human specimens was approved by the local ethic committee on 27.05.2019 (No. 20190527)
13. For publication of patient photos, include a statement confirming that consent to publish was obtained.	not applicable
14. Report any restrictions on the availability (and/or on the use) of human data or samples.	the use of human specimens require a valid ethical consent. Individual patient information cannot be provided publically.
15. Report the clinical trial registration number (at ClinicalTrials.gov or equivalent), where applicable.	not applicable
16. For phase II and III randomized controlled trials, please refer to the CONSORT flow diagram (see link list at top right) and submit the CONSORT checklist (see link list at top right) with your submission. See author guidelines, under 'Reporting Guidelines'. Please confirm you have submitted this list.	not applicable
17. For tumor marker prognostic studies, we recommend that you follow the REMARK reporting guidelines (see link list at top right). See author guidelines, under 'Reporting Guidelines'. Please confirm you have followed these guidelines.	not applicable

F- Data Accessibility

18: Provide a "Data Availability" section at the end of the Materials & Methods, listing the accession codes for data generated in this study and deposited in a public database (e.g. RNA-Seq data: Gene Expression Omnibus GSE39462, Proteomics data: PRIDE PXD000208 etc.) Please refer to our author guidelines for 'Data Deposition'.<br><br>Data deposition in a public repository is mandatory for:<br>a. Protein, DNA and RNA sequences<br>b. Macromolecular structures<br>c. Crystallographic data for small molecules<br>d. Functional genomics data<br>e. Proteomics and molecular interactions	our study does not produce novel Protein, DANN or RNA sequences, Macromolecular structures, crystallographic data, functional genomics data or proteomics and molecular interaction data. All data are available upon request.
19. Deposition is strongly recommended for any datasets that are central and integral to the study; please consider the journal's data policy. If no structured public repository exists for a given data type, we encourage the provision of datasets in the manuscript as a Supplementary Document (see author guidelines under 'Expanded View' or in unstructured repositories such as Dryad (see link list at top right) or Figshare (see link list at top right).	All data required for the evaluation of the study are presented in the manuscript and deposited on a server of the Hans-Knoell Insitute Jena (https://asbdata.hki-jena.de/PressEtAl2021_EMBOMolMed)
20. Access to human clinical and genomic datasets should be provided with as few restrictions as possible while respecting ethical obligations to the patients and relevant medical and legal issues. If practically possible and compatible with the individual consent agreement used in the study, such data should be deposited in one of the major public access-controlled repositories such as dbGAP (see link list at top right) or EGA (see link list at top right).	not applicable
21. Computational models that are central and integral to a study should be shared without restrictions and provided in a machine-readable form. The relevant accession numbers or links should be provided. When possible, standardized format (SBML, CellML) should be used instead of scripts (e.g. MATLAB). Authors are strongly encouraged to follow the MIRIAM guidelines (see link list at top right) and deposit their model in a public database such as Biomodels (see link list at top right) or JWS Online (see link list at top right). If computer source code is provided with the paper, it should be deposited in a public repository or included in supplementary information.	not applicable

G- Dual use research of concern

22. Could your study fall under dual use research restrictions? Please check biosecurity documents (see link list at top right) and list of select agents and toxins (APHIS/CDC) (see link list at top right). According to our biosecurity guidelines, provide a statement only if it could.	We are not aware that our study would as of today fall under dual use restrictions.
